# Climate Action Impacts on Steelmaking Emissions of Persistent Organic Pollutants Highlight a Gap Between the Paris Agreement and the Stockholm Convention

**DOI:** 10.1002/advs.202519769

**Published:** 2026-03-25

**Authors:** Yuxiang Sun, Qiuting Yang, Jianghui Yun, Yujue Yang, Junhao Tang, Qian Liu, Minghui Zheng, Guorui Liu

**Affiliations:** ^1^ College of Geography and Environmental Sciences Zhejiang Normal University Jinhua China; ^2^ State Key Laboratory of Environmental Chemistry and Ecotoxicology Research Center for Eco‐Environmental Sciences Chinese Academy of Sciences Beijing China; ^3^ College of Resource and Environment University of Chinese Academy of Sciences Beijing China; ^4^ School of Environment Hangzhou Institute for Advanced Study UCAS Hangzhou China

**Keywords:** climate change, environmental governance, persistent organic pollutants, public health

## Abstract

Global warming and unintentionally produced persistent organic pollutants (UPOPs) are well‐recognized global concerns. Climate action under the Paris Agreement and UPOPs control under the Stockholm Convention proceed on separate tracks. Evidence of shared health gains is limited. Global steelmaking is a measurable case. Route‐specific UPOPs emission factors and projected changes in output and technology are integrated to estimate 2019–2050 global and regional emissions. Atmospheric and lifetime inhalation‐risk models then quantify added lifetime cancer risk from steelmaking UPOPs. Under climate action, emissions of UPOPs decline by 2050. Yet cancer‐risk inequality persists. Risk shifts from traditional hubs to emerging regions—India, the Middle East, and Africa—where some areas reach 270%–490% of 2019 levels. By 2050, India shows the largest added cancer burden (2201 additional cases). Health‐risk gains are about half the carbon gains; in some regions (e.g., the Middle East, Africa), risk increases even as carbon intensity falls. These findings indicate that current climate action should be complemented by targeted UPOPs controls and a policy bridge between the Stockholm Convention and the Paris Agreement to advance global health and equity. The study provides a data basis for aligning global environmental governance and strategy‐making to advance global health and equity.

## Graphical abstract

Emissions of unintentionally produced persistent organic pollutants (UPOPs) and global warming are two major environmental challenges. But their governance has largely evolved in parallel, leaving the toxicity implications of climate‐driven industrial transitions poorly understood. We quantify how Paris‐aligned steel decarbonization reshapes UPOPs emissions and associated health risks through 2050, providing a scenario‐linked framework to co‐assess CO_2_ mitigation and persistent‐toxics risks and highlighting the need for co‐governance across the Paris Agreement and the Stockholm Convention.

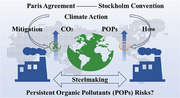

## 1 Introduction

Release of unintentionally produced persistent organic pollutants (UPOPs) [1, 2, 3] and global warming [4, 5, 6] are two of the most pressing environmental threats to human health and planetary stability. UPOPs are known for their environmental persistence, bioaccumulation, long‐range atmospheric transport, and toxicity [7]. They are found in human body [8] and proved to lead severe health effects [9], including cancer, immune system damage, hormone disruption, and developmental disorders. Two governance regimes anchor the global response: UPOPs control under the Stockholm Convention and climate action under the Paris Agreement. Currently, seven UPOPs—polychlorinated dibenzo‐p‐dioxins and furans (PCDD/Fs), polychlorinated biphenyls (PCBs), hexachlorobenzene (HCB), pentachlorobenzene (PeCBz), hexachlorobutadiene (HCBD), and polychlorinated naphthalenes (PCNs)—are listed under Annex C, Part I of the Stockholm Convention, adopted under the auspices of the United Nations Environment Programme (UNEP) and ratified by 186 parties [1]. These UPOPs are regulated and routinely monitored worldwide—for example, through the U.S. Environmental Protection Agency (EPA) Toxics Release Inventory (TRI) [10] and the European Pollutant Release and Transfer Register (EU‐PRTR) [11]. For global warming, the Paris Agreement provides the overarching architecture for climate action [12]. It sets the long‐term temperature goal, operationalized through nationally determined contributions (NDCs) [13] and long‐term low‐emission development strategies (LT‐LEDS) [14] guide structural transitions. In heavy industry, these instruments are translated into sectoral pathway. However, the two governance frameworks are not naturally aligned: climate metrics (CO_2_‐equivalent), reporting cadence (ETF), and compliance logic differ from UPOPs metrics (toxic equivalents, TEQ, an UNEP‐recommended metric that aggregates the toxicity of chlorinated organic pollutants), PRTR/inventory systems, and exposure‐ and health‐based endpoints. How will the climate action affect the global health and equity of unintentional persistent organic pollutant emissions?

Steelmaking sits at the nexus of these twin challenges [15, 16]. Steelmaking is a significant source of UPOPs [17]. Evidence highlights the role of steelmaking in UPOPs emissions. In EU, scrap‐based electric arc furnaces (EAF(Scrap)) and iron ore sintering (IOS) accounted for 17%–27% of total atmospheric emissions of PCDD/Fs and 40%–52% of industrial sources in 2005 [18]. In China, steelmaking contributed 40% of total national atmospheric emissions of PCDD/Fs [19]. Steelmaking processes such as blast furnaces (BF), IOS, and EAF(Scrap) have been explicitly identified in the Toolkit for Identification and Quantification of Dioxin and Furan Releases published by UNEP [17] (referred as Toolkit) as industrial sources of UPOPs. Beyond UPOPs, as one of the most carbon‐intensive industrial sectors, iron and steel production emitted 2507 million tons of CO_2_ in 2019—accounting for 7% of total global emissions and 28% of industrial emissions [20]. To achieve climate goals, the International Energy Agency (IEA) estimates that the steel sector could deliver 6% of the CO_2_ reductions needed by 2050 [21].

Transformation of global steelmaking under the climate action is complex. On the demand side, steel remains an essential material for infrastructure and manufacturing [22]. Steel demand is projected to grow by 30% by 2050 without policy intervention [23]. Strategies such as material efficiency, reuse, and product longevity offer ways to curb this growth [24, 25]. On the supply side, about 70% of global steel is currently produced via the BF‐basic oxygen furnace (BF‐BOF) route [26]. The emergence of circular approaches and low‐carbon steelmaking routes [27, 28] offers significant opportunities to decarbonize this traditional pathway. For example, EAF(Scrap) can reduce CO_2_ emissions by up to 80% compared to BF‐BOF [29]. In recent years, the evolving demand landscape and technological innovation under the climate goal have attracted increasing attention. Studies [30, 31] and international organizations [23, 32, 33] have developed comprehensive and well‐validated transition models for the steel sector, providing detailed and systematic pathways toward net‐zero emissions. However, growing evidence indicates that certain steelmaking technologies may unintentionally exacerbate emissions of UPOPs [16, 34, 35, 36]. EAF(Scrap), for example, while beneficial for CO_2_ mitigation, is increasingly recognized as a hotspot for UPOPs release [16]. Additionally, UPOPs emissions from new steelmaking technologies remain poorly understood. To date, UPOPs emission assessments in steelmaking remain confined to isolated process stages, lacking integration across the full production chain [37, 38, 39]. Well‐developed models for decarbonization in the steel sector are largely disconnected from emissions of UPOPs and associated health risks. The temporal evolution and spatial distribution of atmospheric emissions and related health risks of UPOPs from global steelmaking under climate scenarios remain absent. This lack of comprehensive evidence constrains the ability to foster the deep integration and co‐benefits of pollution control and climate governance.

In this study, we complied route‐ and stage‐specific emission factors for UPOPs regulated by Stockholm Convention in steelmaking (Table S1). Combined with trajectories of steelmaking under base and climate scenarios, an emission‐factor approach is adapted to estimate global and regional UPOPs emissions and their structural evolution by 2050. Health risk in each steel producing region resulted by UPOPs emissions is assessed by a multimedia steady‐state atmospheric model with a lifetime inhalation risk model. Additional cancer cases are estimated by integrating scenario‐consistent, age‐structured population trajectories and cancer risks. Finally, we compared the reduction in cancer burden per unit of steel output with the reduction in carbon intensity, quantifying health co‐benefits of climate action and highlighting potential inequalities across regions. The results provide quantitative support for coordinating UPOPs and greenhouse‐gas governance frameworks and to advance global health and equity.

## 2 Results

### 2.1 Emission of UPOPs From Steelmaking

**FIGURE 1:**
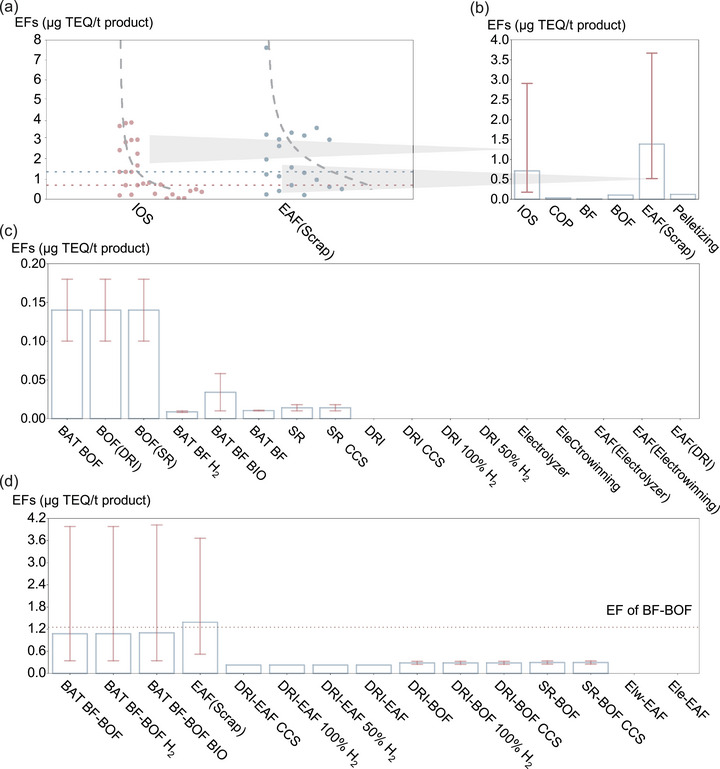
Emission factors (EFs, toxic equivalent emitted per tonne of product (TEQ/t)) of polychlorinated dibenzo‐p‐dioxins and dibenzofurans (PCDD/Fs) from steelmaking processes and routes. (a) Reported EFs of scrap‐based electric arc furnace steelmaking (EAF(Scrap)) and iron ore sintering (IOS) stages based on all available site‐specific measurements (Probability distribution fitting of PCDD/Fs emission factors for EAF(Scrap) (n = 19) and IOS (n = 29) was performed in log space using RStudio (v2025.09.2) and evaluated with the Kolmogorov–Smirnov test with p > 0.05); (b) EFs of PCDD/Fs associated with key stages of existing steelmaking processes (error bars denote the log‐normal one‐standard‐deviation range); (c) Estimated EF ranges of PCDD/Fs for emerging low‐carbon steelmaking stages, derived from a mechanism‐based assessment framework (error bars denote the lower and upper bounds of emission factors derived from the mechanism‐based assessment); and (d) PCDD/Fs EFs for complete steel production routes, including both conventional and emerging pathways (error bars denote the propagated route‐level variability range derived from the stage‐level ranges in panels (b) and (c)). (BF: blast furnace ironmaking; BOF: basic oxygen furnace steelmaking; Pelletizing: iron ore pelletizing; BAT BOF: BOF with best available technology; BOF (DRI): BOF fed by reduced iron; BAT BF: BF with best available technology; BAT BF H_2_: BAT BF with hydrogen injection; BAT BF BIO: BAT BF with biomass‐based reductant substitution; SR: smelting reduction ironmaking; SR CCS: SR with carbon capture and storage; DRI: direct reduced iron; DRI CCS: DRI with CCS; DRI 100% H_2_: DRI using 100% hydrogen as reductant; DRI 50% H_2_: DRI with 50% hydrogen as reductant; Electrolyzer: electrolytic ironmaking; Electrowinning: electrowinning‐based ironmaking; EAF (Electrolyzer): EAF fed by electrolytic iron; EAF (Electrowinning): EAF fed by electrowon iron; EAF (DRI): EAF fed by DRI; Elw‐EAF: Electrowinning‐Electric Arc Furnace steelmaking route; Ele‐EAF: Electrolysis‐EAF route. Detailed information about steelmaking routes could be seen in Table S3).

EAF(Scrap) and IOS are acknowledged as the primary sources of UPOPs in steelmaking [17, 40, 41]. As shown in Figure 1a, EAF(Scrap) contributes more significantly to atmospheric emissions of PCDD/Fs than IOS with emission factor at 1.384 (0.522–3.666))  µg TEQ·t^−1^ product, nearly double that of IOS (0.708 (0.172–2.91) µg TEQ·t^−1^, Figure 1b). EAF(Scrap) also exhibit higher emission factors for other UPOPs (Table S2) among all steelmaking processes, including PCBs (0.173 (0.053–0.564) µg TEQ·t^−1^), HCB (2000 µg·t^−1^), PeCBz (1200 µg·t^−1^)) and PCNs (0.026 (0.020–0.031) µg TEQ·t^−1^), compared with IOS values of 0.033 (0.003–0.324) µg TEQ·t^−1^, 327 (177–600) µg·t^−1^, 1159 (1015–1324) µg·t^−1^ and 0.004 (0.001–0.012) µg TEQ·t^−1^, respectively. Pelletizing has the emission factor of PCDD/Fs of 0.12 µg TEQ·t^−1^, slightly higher than the 0.1 µg TEQ·t^−1^ reported for BOF in the UNEP Toolkit [1]. Emissions from BF and coke production (COP) stages are lower, with PCDD/Fs emission factors of 0.01 and 0.03 µg TEQ·t^−1^, respectively. For other UPOPs, BF, COP, and BOF stages remain minor contributors: emissions of PCBs and PCNs typically range from 0.001 to 0.002 µg TEQ·t^−1^, while HCB and PeCBz emissions are approximately 1–2 µg·t^−1^. HCBD has much lower emission factors for all processes. For subsequent calculations, we used the central estimate of emission factors. Results calculated using the lower and upper bounds are reported in Data S1–S4.

Emerging low‐carbon steelmaking technologies (Table S3) can be categorized into five main types: (i) modified BF–BOF, (ii) direct reduced iron (DRI)–EAF using alternative reductants, (iii) smelting reduction (SR)‐BOF, (iv) electrolytic routes, and (v) carbon capture and storage (CCS)‐integrated processes. Unlike CO_2_, UPOPs are nonpolar, hydrophobic, and exhibit low volatility [42], meaning that standard CO_2_ capture technologies—such as amine‐based solvents [43] or cryogenic separation [44]—are ineffective for UPOPs removal. In best available technique‐level (BAT) BF, partial coke substitution with biochar can elevate chlorine input [45] (Text S1), leading to PCDD/Fs emission factors of up to 0.034 µg TEQ·t^−1^ (Figure 1c). SR, operating above 1000°C in vessels (Figure S1), exclusively use pulverized coal as a reductant, which elevates chlorine input and leads to PCDD/Fs emission factor up to 0.018 µg TEQ·t^−1^. In BAT BOF, higher scrap ratios may introduce chlorine impurities, with emission factor reaching 0.14 µg TEQ·t^−1^. By contrast, DRI processes using natural gas or H_2_ exhibit negligible UPOPs formation due to chlorine‐free reductants and highly reducing conditions. Similarly, electrowinning (Elw) [46] and electrolyzer‐based (Ele) systems [47] operate outside the critical de novo synthesis window and lack chlorine precursors, yielding minimal UPOPs emissions. Downstream use of DRI or electrolytic products in EAF does not reintroduce significant risk due to the absence of favorable chemical and thermal conditions. For other UPOPs, the variation of emission factors across processes follows a pattern similar to that of PCDD/Fs.

Mass flow‐adjusted analysis indicates that EAF(Scrap) and BF‐BOF dominate PCDD/Fs (Figure 1d) and other UPOPs emissions (Table S4) among low‐carbon steelmaking routes. Emission factors of HCB, PCBs, and PCNs for EAF(Scrap) are 3–4 times higher than these of BF‐BOF, while these of PCDD/Fs, PeCBz, and HCBD remain consistent across these two routes. For emerging steelmaking routes, although BAT BF‐BOF with hydrogen or biochar injection (BAT BF‐BOF H_2_ and BAT BF‐BOF BIO, respectively) offer moderate improvements, their emission reductions of PCDD/Fs are generally limited. DRI‐EAF‐based steelmaking routes demonstrate lower emission factor of UPOPs. DRI‐BOF and SR‐BOF‐based routes exhibit comparable emissions, close to that of DRI‐EAF. For other UPOPs, emission factors for BAT BF‐BOF are comparable to those of BF‐BOF.

### 2.2 Global Emissions of UPOPs Under the Climate Actions

**FIGURE 2:**
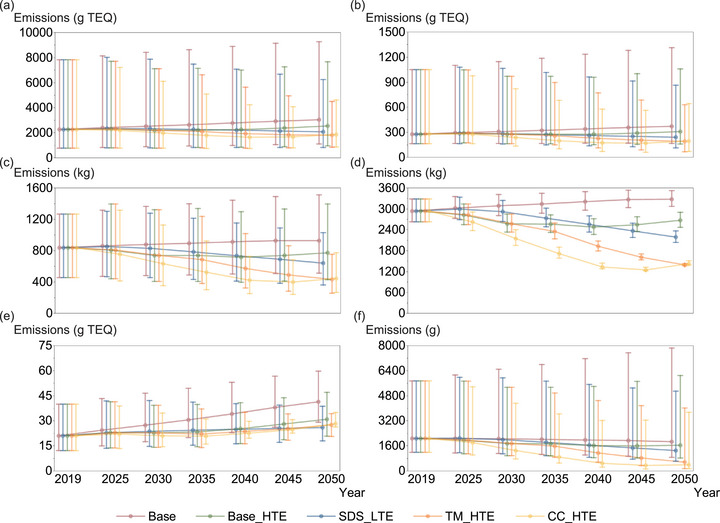
Global emissions of (a) polychlorinated dibenzo‐p‐dioxins and dibenzofurans (PCDD/Fs) (g toxic equivalent (g TEQ)), (b) polychlorinated biphenyls (PCBs) (g TEQ), (c) hexachlorobenzene (HCB) (kg), (d) pentachlorobenzene (PeCBz) (kg), (e) polychlorinated naphthalenes (PCNs) (g TEQ), and (f) hexachlorobutadiene (HCBD) (g) from 2019 to 2050 under Base, Base_HTE, SDS_LTE, TM_HTE and CC_HTE scenarios. Scenario details are provided in Section 4.3. (Error bars denote the lower‐upper range of emission estimates arising from route‐level emission‐factor variability.).

Global emissions of UPOPs rise sharply under the no‐additional‐policy Base scenario (Figure 2). By 2050, projected emissions reach 3039 g TEQ, 370 g TEQ, 929 kg, 3280 kg, 41 g TEQ, and 1862 g for PCDD/Fs, PCBs, HCB, PeCBz, PCNs, and HCBD, respectively. Except for HCBD, 2050 emissions of the other five UPOPs increase by 11%–97% relative to 2019 (Figure 2a–f), with the largest gains in PCDD/Fs, HCB, and PeCBz. With higher technology expectations in the baseline (Base_HTE), innovation partially dampens emission growth. By 2050, PCDD/Fs, PCBs, and PCNs rise by 12%–47% from 2019, while the other pollutants decline.

Climate action curbs growth more decisively. In a sustainable pathway with slower technology penetration (71% from EAF(Scrap) and BF‐BOF based steelmaking routes in 2050) but stronger recycling and demand management (SDS_LTE) with 20% reduction in steel production compared with Base in 2050, total UPOP emissions fall by 30%–37% versus the baseline, and five species—all except PCNs—drop below their 2019 levels. Scenarios assuming rapid technological progress coupled with targeted policy instruments, such as suspending carbon‐intensive technologies (TM_HTE) or imposing high carbon cost (CC_HTE), drive deeper transformations, cutting emissions by more than 40% relative to the baseline by 2050. Under these conditions, PCDD/Fs and PCBs decline to about 70%–80% of their 2019 levels, while HCB and PeCBz fall to roughly 50%.

### 2.3 Regional Emissions of UPOPs Under the Climate Actions

**FIGURE 3:**
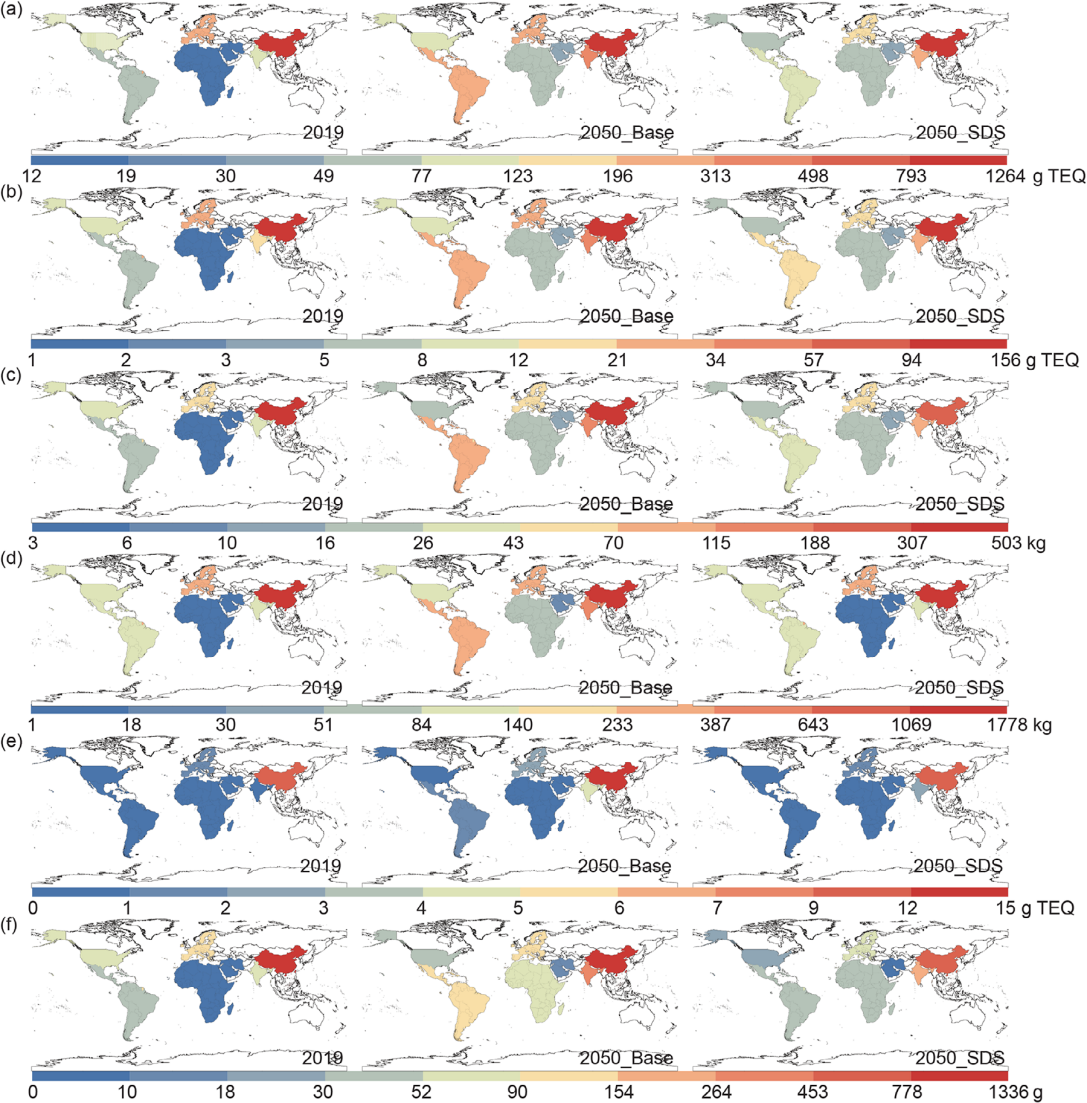
Regional emissions of (a) polychlorinated dibenzo‐p‐dioxins and dibenzofurans (PCDD/Fs) (g toxic equivalent (g TEQ)), (b) polychlorinated biphenyls (PCBs) (g TEQ), (c) hexachlorobenzene (HCB) (kg), (d) pentachlorobenzene (PeCBz) (kg), (e) polychlorinated naphthalenes (PCNs) (g TEQ), and (f) hexachlorobutadiene (HCBD) (g) from the steel sector in 2019 and 2050 under Base and SDS_LTE scenarios. Scenario details are provided in Section 4.3.

Global emission of UPOPs (Figure 3a–f) are modeled under the Base and SDS_LTE scenarios. China and EU are currently the dominant sources of UPOPs from global steelmaking. In 2019, China accounted for 60%–80% of emissions across major producing regions, depending on pollutant. Its steel sector released 1264 g TEQ of PCDD/Fs and 156 g TEQ of PCBs—73% of emissions from major regions—while HCB and PeCBz each exceeded 75%. EU ranked second with about 10% of global emissions, and each of the remaining regions contributed only 1%–6%. In the Base scenario, the relocation of steel capacity reshapes regional patterns. By 2050, China's annual emissions fall relative to 2019 (HCB and PeCBz by 20%, PCDD/Fs and PCBs by 6%), yet China still accounts for 50% of the total, remaining the largest emitter. By contrast, emissions in emerging and developing regions surge, rising to 2–7 times their 2019 levels. India emerges as a major contributor, with its share of the six UPOPs surpassing 20%. Central and South America rise to 10%, comparable to EU.

Climate action moderates these trends. Under SDS_LTE, India and Central and South America see the steepest reductions (40%–50% below baseline), followed by China (30%–40%) and EU/United States (20%–30%). In the Middle East, however, emissions remain close to baseline, with PCDD/Fs even slightly higher. The global spatial pattern of emissions nonetheless persists: as new steel capacity concentrates in developing regions, their UPOPs emissions continue to grow. Relative to 2019, 2050 emissions under SDS_LTE remain 50%–65% higher in Central and South America, 100%–200% higher in India and the Middle East, and 100%–500% higher in Africa, while traditional production regions—China, the EU, and the United States—see reductions exceeding 20%.

### 2.4 Global and Regional Evolution of Toxic Emission Structures

**FIGURE 4:**
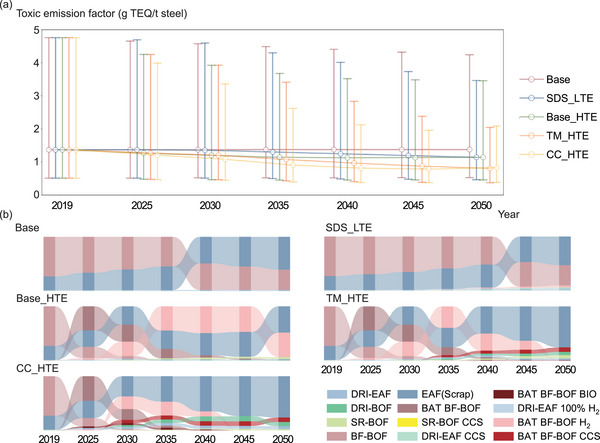
Global toxic emission structures. (a) Toxic equivalent emissions per unit of crude steel production across scenarios (g TEQ/t steel) (Error bars denote the lower‐upper range of estimates arising from route‐level emission‐factor variability); and (b) composition of unintentionally produced persistent organic pollutants emissions by production route under different scenarios. Scenario details are provided in Section 4.3.

To allow comparison across pollutants, total emissions of all UPOPs were expressed in terms of TEQ (toxic emissions). The conversion employed pollutant‐specific inhalation unit risks, defined as the probability of developing cancer from continuous lifetime exposure (assumed 70 years) to an atmospheric concentration of 1 µg·m^−^^3^ of the substance [48, 49]. In the Base scenario, the cost advantage of EAF(Scrap) drives a shift away from BF‐BOF, increasing reliance on this higher‐intensity process. By 2050, global average toxic emission factor reaches 1.37 µg TEQ t^−^^1^ (Figure 4a), with EAF(Scrap) responsible for 61% of emissions and BF‐BOF for 38% (Figure 4b). In Base_HTE, wider deployment of BAT on the BF‐BOF route lowers the emission factor by 17% relative to Base, even in the absence of additional climate policy. In SDS_LTE, enhanced material circularity expands the share of EAF(Scrap), but wider diffusion of lower‐intensity routes (DRI–EAF and SR‐BOF) holds 2050 intensity at a level comparable to Base_HTE. In this scenario, EAF(Scrap) and BF‐BOF contribute 53% and 37% of emissions, respectively. Under TM_HTE and CC_HTE, stringent climate measures accelerate technology transformation: while EAF(Scrap) remains the main emitting route (76%), the forced penetration of other low‐carbon processes lowers global 2050 intensity by 41% relative to Base.

(BF‐BOF: blast furnace ironmaking‐basic oxygen furnace steelmaking route; EAF (Scrap): scrap‐based electric arc furnace steelmaking route; DRI‐EAF: Direct‐reduced‐iron‐fed EAF route; DRI‐BOF: DRI‐fed BOF route; SR‐BOF: Smelting reduction ironmaking‐BOF route; SR‐BOF CCS: SR‐BOF route with carbon capture and storage; DRI‐EAF CCS: DRI‐EAF route with CCS; DRI‐EAF 100% H_2_: DRI‐EAF route using 100% hydrogen as the reductant; BAT BF‐BOF: BF‐BOF route with best available technology; BAT BF‐BOF CCS: BAT BF‐BOF route with CCS; BAT BF‐BOF H_2_: BAT BF‐BOF route with hydrogen injection; BAT BF‐BOF BIO: BAT BF‐BOF route with biomass‐based reductant substitution).

**FIGURE 5:**
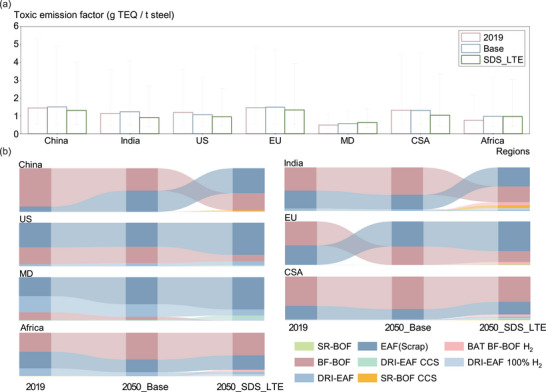
Regional toxic emission structures. (a) Toxic equivalent emissions per unit of crude steel production across scenarios (g TEQ/t steel) (Error bars denote the lower‐upper range of estimates arising from route‐level emission‐factor variability); and (b) composition of unintentionally produced persistent organic pollutant emissions by production route in 2019, 2050 under SDS_LTE and Base scenarios. Scenario details are provided in Section 4.3.

By region, China and EU recorded the highest toxic emission factors in 2019, both at 1.45 µg TEQ t^−^^1^ (Figure 5). Production in these regions was dominated by BF‐BOF and EAF(Scrap), making them the principal emitting routes; Central and South America displayed a similar profile. In contrast, the United States and the Middle East exhibited lower factors due to the prevalence of natural‐gas‐based DRI, with the Middle East registering the lowest global level (0.48 µg TEQ t^−^^1^). India and Africa also reported comparatively low factors in 2019, reflecting the contribution of newer‐capacity installations. In the Base scenario, China and EU shift from BF‐BOF dominance to a more balanced BF‐BOF/EAF(Scrap) mix, yet their 2050 toxic emission factors exceed 2019 values. In India, Africa, and the Middle East, limited policy constraints steer new capacity toward BF‐BOF and EAF(Scrap), increasing emission factors by 10%–30%. Under SDS_LTE, climate action accelerates adoption of EAF(Scrap) and DRI–EAF. The diffusion of these lower‐intensity routes enables China, EU, India, and Central and South America to achieve 10%–20% reductions in 2050 factors relative to 2019. Nonetheless, the scale of production in China and India continues to necessitate BF‐BOF, keeping BF‐BOF and EAF(Scrap) as the main emitting processes. In EU and the United States, EAF(Scrap) becomes the dominant source, while in the Middle East the 2050 factor rises by 10% relative to Base, driven mainly by further expansion of EAF(Scrap).

(EU: European Union; CAS: Central and South America; US: the United States; ME: Middle East; BF‐BOF: blast furnace ironmaking‐basic oxygen furnace steelmaking route; EAF (Scrap): scrap‐based electric arc furnace steelmaking route; DRI‐EAF: Direct‐reduced‐iron‐fed EAF route; SR‐BOF: Smelting reduction ironmaking‐BOF route; SR‐BOF CCS: SR‐BOF route with carbon capture and storage; DRI‐EAF CCS: DRI‐EAF route with CCS; DRI‐EAF 100% H_2_: DRI‐EAF route using 100% hydrogen as the reductant; BAT BF‐BOF H_2_: BAT BF‐BOF route with hydrogen injection).

## 3 Discussion

### 3.1 Climate Action Impacts on Global High‐Risk Regions and Vulnerable Populations

**FIGURE 6:**
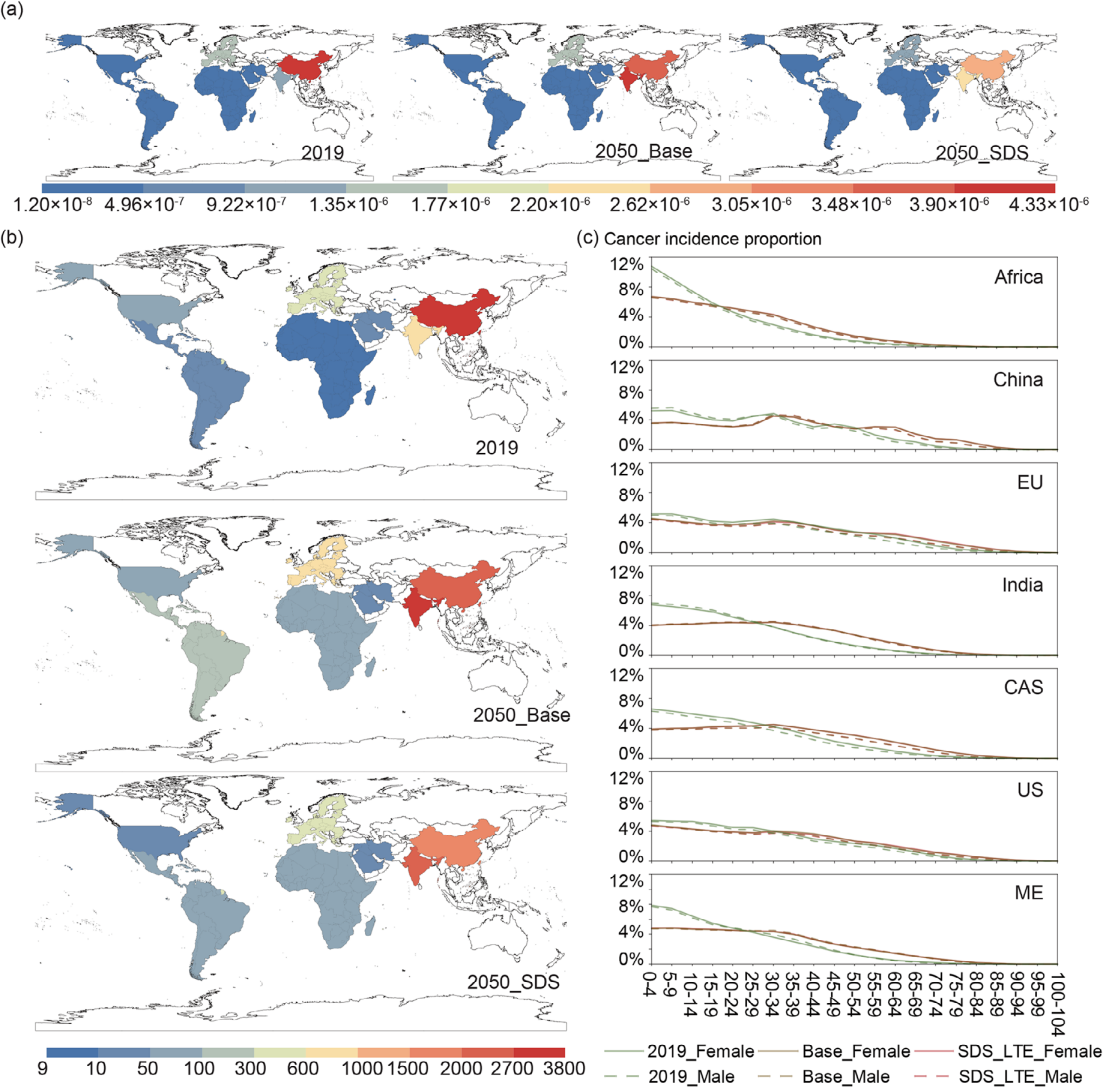
Regional inhalation cancer risks and cancer cases caused by unintentionally produced persistent organic pollutant emissions from steelmaking. (a) Regional lifetime inhalation exposure risk (unitless; expressed as probability), (b) regional cancer cases caused by lifetime inhalation, and (c) age‐group share of lifetime inhalation‐attributable cancer cases shown separately for females and males in 2019 and 2050 under Base and SDS_LTE scenarios. Scenario details are provided in Section 4.3.

The risk model, grounded in UPOPs emissions, reveals stark regional disparities in population health risks. In 2019, toxic emissions of UPOPs from steel sector in China, EU and India produced the incremental lifetime inhalation cancer risk exceeding the U.S. EPA's de minimis threshold [50] of 1 × 10^−^^6^ (Figure 6a). Under the Base scenario, by 2050 cancer risks in China the United States remain unchanged despite of the decline of productions, while EU shows a slight increase. In contrast, risks escalate sharply in other major producing regions. The cancer risk increase for the Middle East, Central and South America, India, and Africa by 2050 are 160%, 220%, 360%, and 560% of their 2019 levels, respectively. By 2050, India becomes the highest‐risk region (4.33 × 10^−^^6^), while China and EU remains above the de minimis threshold. Climate action constrains the growth of steel‐related risks. Under SDS_LTE, incremental risks in China, EU, and the United States fall by 16%–33% relative to 2019. Yet the inequality pattern persists: risks in developing and less‐developed regions continue to climb, with India reaching 270% and Africa 490% of their 2019 levels. The Middle East shows an anomaly, with 2050 risks under SDS_LTE unchanged compared with those in the Base scenario.

(2019_Female: proportion for female in 2019; Base_Female: proportion for female in 2050 under Base scenario; SDS_LTE_Female: proportion for female in 2050 under SDS_LTE scenario; 2019_Male: proportion for male in 2019; Base_Male: proportion for male in 2050 under Base scenario; SDS_LTE_Male: proportion for male in 2050 under SDS_LTE scenario; EU: European Union; CAS: Central and South America; US: the United States; ME: Middle East).

Incorporating population structure and survival trajectories (Data S5) from the Wittgenstein Centre's database, and combining age‐sex distributions with life expectancy [51], long‐term excess cancer cases attributable to steel emissions are estimated (Figure 6b,c). In 2019, China contributed 3158 additional cases, India 795, EU 548, and all other regions fewer than 100. By 2050 (Base), India records the largest burden (3719 cases), while EU, the Middle East, Central and South America, and Africa each rise to several tens to several hundreds of cases. In contrast, China declines to 2554. Under SDS_LTE, the burden is reduced but not eliminated: 2201 cases in India, 1751 in China, and 472 in EU—all still higher than in 2019. Africa, the Middle East, and Central and South America maintain steep upward trajectories, underscoring how even with climate action, developing regions and key suppliers will continue to shoulder disproportionate health burdens.

By gender, females consistently exhibit slightly higher case increments than males because of longer life expectancy and greater cumulative exposure. By age, in 2019 excess cases were concentrated among younger cohorts, owing to high birth rates, a large youth base, and longer residual lifespans. By 2050 (Base), Africa continues to show the highest incidence in younger populations, while India, the Middle East, and Central and South America shift toward both younger and middle‐aged cohorts. China, EU and the United States retain balanced age distributions. Under SDS_LTE, demographic dynamics reinforce this trend, with middle‐aged groups emerging as the main risk‐bearers.

### 3.2 Climate Action Impacts on Regional Inequalities in Health Burdens and Co‐Mitigation Benefits

**FIGURE 7:**
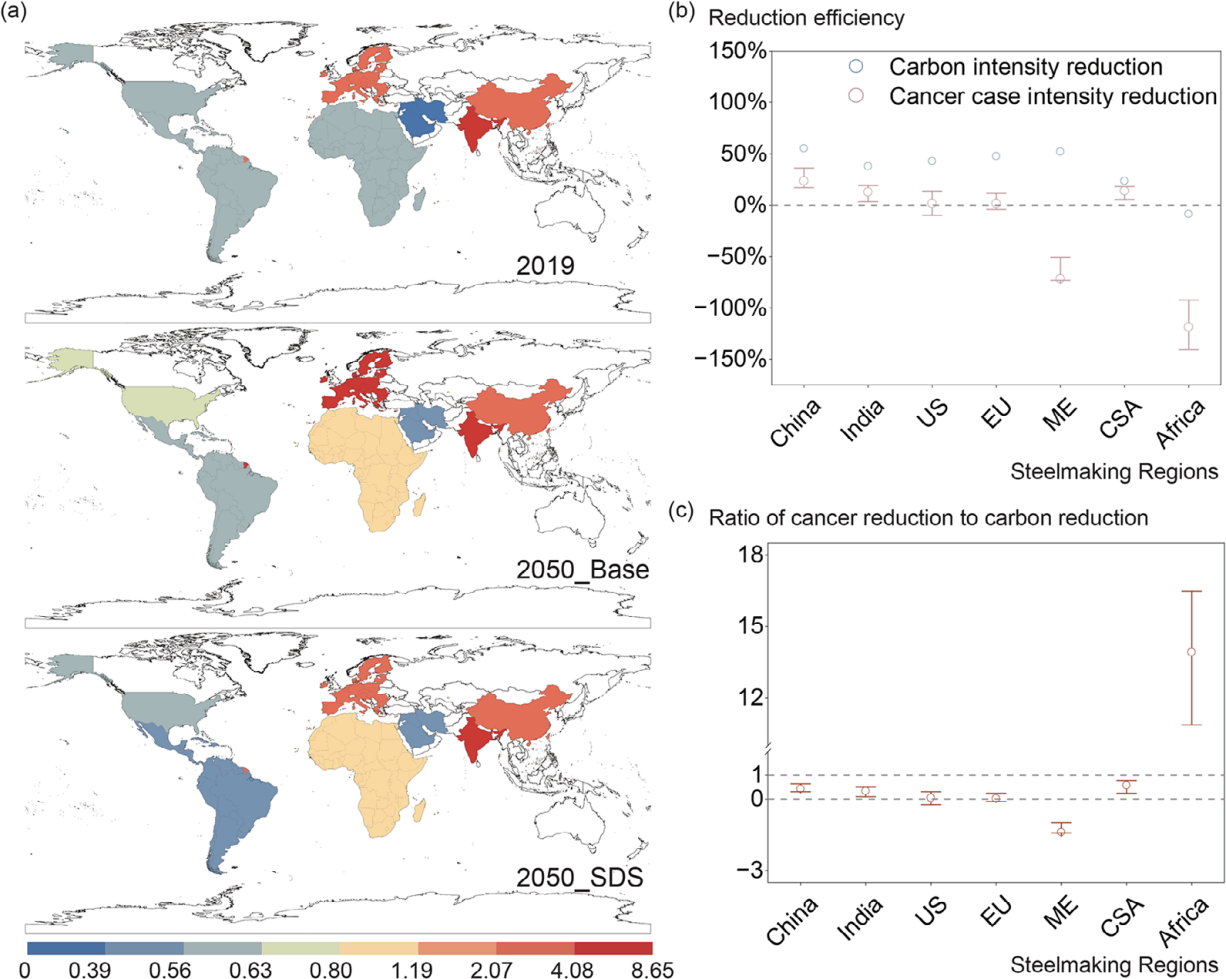
Health burdens and co‐mitigation benefits of unintentionally produced persistent organic pollutant emission from steelmaking under climate action (a) Number of cancer cases caused by lifetime inhalation per million steel production in each region, (b) reduction of carbon intensity and cancer cases intensity from 2019 to 2050 under SDS_LTE, (Error bars denote the lower‐upper range of efficiency estimates arising from route‐level emission‐factor variability) and (c) ratio of unit cancer case reduction to unit carbon emission reduction (Error bars denote the lower‐upper range of ratio estimates arising from route‐level emission‐factor variability). Scenario details are provided in Section 4.3.

Beyond absolute increases, the unit health burdens, represented as number of cancer cases caused by lifetime inhalation per unit of steel production, is projected to become more uneven across regions. In 2019, India, EU, and China bore the highest unit health burdens. Under the Base scenario, China's unit health burden declines, while India and EU continue to rise, reaching 8.65 and 4.23 cases per million tonnes, respectively (Figure 7a). With climate action, overall health burdens fall in the SDS‐LTE scenario, but disparities persist. By 2050, India's unit health burden climbs to 6.26 cases per million tonnes—almost three times that of China—despite similar total case numbers, making it the highest worldwide. EU records fewer absolute cases than China, but maintains the second‐highest unit health burden globally (3.44). Emerging producers such as Africa and the Middle East, though starting from low baselines, experience sharp increases of 120% and 70%, respectively. Overall, climate action reduces absolute burdens but does not eliminate stark regional inequalities in health impacts of steel production.

(EU: European Union; CAS: Central and South America; US: the United States; ME: Middle East).>

A further comparison underscores that the co‐benefits of climate action for UPOP‐related health risks lag well behind those for carbon mitigation. In the SDS‐LTE scenario, by 2050 reduction in unit carbon emission for steelmaking exceeds 50% in China, India, the United States, and EU, yet reductions in expected cancer cases per unit of steel are only 24%, 12%, 2%, and 1%, respectively (Figure 7b). The Middle East presents a paradox: unit carbon emissions fall by 52%, while health burdens rise by 70%. Africa shows the sharpest mismatch, with a 9% increase in carbon emissions but a 120% surge in unit health burden. By contrast, Central and South America demonstrates the most favorable “carbon–health synergy”: reductions in cancer risk per unit of steel reach 60% of the carbon mitigation level, compared with 40% in China, 30% in India, and <10% in EU and the United States (Figure 7c).

### 3.3 Drivers of Global and Regional UPOPs Emissions and Associated Health Risk

Climate action is reshaping the global landscape of steelmaking UPOPs emissions, the regional distribution of risks, and the structure of vulnerable populations. Combined effects of activity changes, social conditions, and climate‐driven changes in UPOPs environmental behavior could cause the global emission changes and regional risk divergences.

From a global perspective, climate‐target‐driven demand‐side optimization create a positive scale effect for reducing UPOPs emissions. Yet, at the technology level, the drivers are more complex. DRI‐ and SR‐based routes can offer carbon–toxicant co‐benefits (Table S5), while their large‐scale deployment remains constrained by real‐world barriers such as costs, energy requirements, and infrastructure readiness. Meanwhile, economic and material realities continue to favor scrap‐based EAF in many regions. By 2030, the cost of EAF(Scrap) steel is projected to be USD 371–401 per tonne [33]—significantly lower than BAT BF–BOF, DRI–EAF, or SR–BOF. Even with falling hydrogen costs, the economic advantage of EAF(Scrap) is expected to persist. Meanwhile, global in‐use steel stocks have already exceeded 30 billion tonnes and continue to grow [26], making EAF(Scrap) the primary pathway to low‐carbon steel. In line with this, it is (Figure 1) proved that UPOPs emission intensity in EAF‐based routes can be higher than that of BF‐BOF for certain UPOPs categories, implying that technology shifts may generate mixed effects. Across the three climate‐target scenarios (SDS_LTE, TM_HTE, CC_HTE), the EAF(Scrap) share increases from 20% in 2019 to 38%–40% in 2050 [23, 33]. But in scenarios with higher technology expectations (TM_HTE and CC_HTE), the penetration of routes with low UPOPs emissions such as DRI‐EAF generally remains below 20%. Therefore, global UPOPs changes reflect a combined effect of reducing emissions from capacity contraction and offsetting part of the reductions from EAF(Scrap) expansion.

Regional differences in emissions and risks in our study are mainly governed by regional redistribution of output and capacity. China provides a clear example. In 2019, BF‐BOF accounted for 89% of China's steel production, while the remainder relied on EAF(Scrap) [52]. Due to the lock‐in of existing assets, high decommissioning costs, and industrial inertia, complete near‐term phase‐out of BF‐BOF is difficult. At the same time, growing societal scrap availability and make EAF(Scrap) one of China's key transition routes. As a result, even if China's total production declines in climate‐target scenarios, the rising share of EAF(Scrap) (50% under SDS_LTE in 2050) can limit the magnitude of risk reductions relative to the baseline. EU illustrates a different constraint profile. Its current steel production relies on both BF‐BOF and EAF(Scrap) to a comparable extent, while further expansion of EAF(Scrap) faces constraints related to scrap availability [53] and quality structure [23]. Decarbonization depends, to a limited extent, on the deployment of DRI‐EAF H_2_ (10% under SDS_LTE in 2050). As a result, the potential for additional UPOPs reductions remains limited. In India, Africa, Central and South America, and the Middle East, demand growth and capacity expansion are more pronounced under SDS_LTE with 2050 output potentially reaching 2–4 times of the 2019 level. The scale effect alone can increase emissions and risks. For India and Central and South America, rapid capacity expansion is accompanied by increasing shares of DRI‐EAF and SR‐BOF, which can partially offset the risks associated with output relocation. In the Middle East, DRI‐EAF account for 70% of steel production in 2019 because of rich natural‐gas resources. However, as capacity expands in climate‐target scenarios, an increasing role of EAF(Scrap) could reintroduce UPOPs pressures. In Africa, incremental production still relies heavily on BF‐BOF and EAF(Scrap). The combination of more output and higher shares of high‐UPOPs routes can amplify regional emission and risk burdens.

Beyond emissions, climate targets can also reshape inequalities through demographic evolution. Within regions, vulnerability differs by gender (e.g., longer female lifespans imply greater cumulative exposure) and by age (larger youth and middle‐aged cohorts imply longer exposure horizons), extending inequality from “between regions” to “within populations.” UNICEF projections indicate that by 2050, around 40% of all births will be in Africa [54], and India will remain one of the world's largest child populations by 2050 [55]. In contrast, China, Europe, and the United States are expected to experience population ageing and slower growth [51]. Given the persistence and cumulative exposure potential of UPOPs, younger populations in Africa and India may face higher cumulative‐risk potential from both the increasing emissions and population structure.

In addition, other factors could drive the regional differences. IPCC AR6 emphasizes that climate‐related risks often disproportionately affect marginalized groups and vulnerable regions, reflecting uneven capacities in regulation, enforcement, occupational protection, healthcare access, and public‐health infrastructure [56]. The exposure‐to‐risk translation may be higher in settings with weaker protective systems, concentrating health burdens. From the perspective of environmental conditions, warming can influence steady‐state concentrations and exposure assessments by altering key atmospheric processes of UPOPs. A detailed discussion of temperature perturbations and associated parameter changes in Text S2. However, due to limited evidence across regions, the contribution of the socioeconomic factors or warming‐induced perturbations to regional differences currently lacks consistent and high‐quality data. Quantitative research and field studies are needed to better constrain region‐specific parameter responses and improve attribution of their contribution to future UPOPs risks.

### 3.4 Co‐Management of Climate and UPOPs Health Risks

From a technological perspective, Evidence shows that during preheating (200°C–700°C) [57], organic coatings and impurities on scrap surfaces readily generate complex UPOPs [58]. Effective suppression strategies include rigorous scrap quality sorting and pretreatment, as well as optimized thermal management during preheating to limit precursor availability and suppress reaction kinetics. In addition, developing regions continue to rely on BF‐BOF to host new capacity expansion [23]. Mechanistically, within iron and steel processes (especially IOS and BF‐BOF systems), PCDD/Fs are primarily generated within the 250°C–500°C, facilitated by carbonaceous residues [59], chlorine [60, 61, 62, 63], and metal catalysts (e.g., Fe, Cu [64, 65, 66]). Viable suppression strategies include reducing chlorine‐ and carbon‐rich inputs, shortening residence time in the critical window, applying flue gas recirculation to lower oxygen supply [67, 68], and partially replacing pulverized coal injection with hydrogen‐rich fuels [69], which both reduce chlorine/carbon input and scavenge Cl· radicals via H·, interrupting PCDD/F formation [70]. More broadly, the UPOPs emission profiles of new processes require more comprehensive, time‐sensitive monitoring and mechanistic identification to avoid unintended low‐carbon but high‐toxic lock‐ins.

From a risk and population protection perspective, model results indicate regional health risks are uneven and persistent. Risks have partially subsided in traditional hubs but are being redistributed to emerging regions through capacity and process shifts, such as Africa and the Middle East. In these regions, women and children may face disproportionately higher health risks due to longer cumulative exposure windows and heightened vulnerability, while multiple coexisting challenges—such as limited access to advanced pollution‐control technologies and constrained healthcare capacity—can further amplify health burdens. Monitoring networks and pilot projects should be prioritized in high‐risk regions, focus industrial efforts on priority processes such as BF‐BOF and EAF(Scrap), and design health protection and compensation mechanisms for vulnerable groups.

From a governance perspective, global UPOPs control in the steel sector significantly lags behind carbon reduction. This lag arises from multiple sources: (i) an instrumental lag—long‐term strategies and pathways in the steel industry remain CO_2_‐centric, with inadequate integration of UPOPs constraints [23, 25, 28]; (ii) an observational lag—UPOPs monitoring, inventories, and exposure–health evidence take longer to build and lack sufficient spatial coverage [17, 71], unlike the high‐frequency monitoring–reporting–verification (MRV) systems [72] for greenhouse gases; (iii) a technological lag—dedicated UPOPs control technologies are less developed compared with decarbonization technologies; and (iv) institutional misalignment—the UNFCCC/Paris Agreement [12] and the Stockholm Convention currently operate with different targets, metrics, and compliance frameworks. Explicitly incorporating UPOPs constraints and establishing dual “carbon–health” performance frameworks to continuously track co‐benefits, are critical. Strengthening linkages between the Paris Agreement and the Stockholm Convention—such as exploring MRV–PRTR [73] data interoperability and embedding UPOPs information into low‐carbon transition monitoring—would help bridge the gap. Meanwhile, capacity building and technology transfer in high‐growth regions (India, Africa, Middle East) are essential. Positioning carbon reduction, toxicant reduction, and inequality reduction as parallel policy goals, underpinned by verifiable indicators, will help translate aggregate climate gains into more equitable environmental health benefits.

## 4 Methods

### 4.1 Emission Factor Data

To derive emission factors for various steelmaking routes, reported atmospheric UPOPs emission levels from different production stages were integrated with corresponding material flows. For processes with available emission factor data, a tiered approach was used to ensure consistency, transparency, and reliability: (1) UNEP‐authoritative sources: where available, emission factors defined in UNEP's Toolkit [17] were prioritized; (2) UNEP‐authoritative sources: where applicable, emission factors from official inventories—such as those by the EEA [41]—were adopted; (3) Peer‐reviewed studies and regional programs: for processes not covered by formal inventories, data were compiled from peer‐reviewed literature and programs such as those by the European Commission. In total, over 100 site‐based measurements from steel plants in more than 20 countries and regions, spanning the past two decades, were synthesized (Table S1). This provides broad temporal and geographical coverage to support the robustness and representativeness of the compiled emission factors. Derivation of emission factor followed standardized procedures outlined by EPA and UNEP's Toolkit methodologies. Distributions of emission‐factor data were statistically fitted (Figures S2 and S3). Emission factor data were lognormally distributed (Figures S2 and S3), and the geometric means were calculated in log space and back‐transformed to the original scale, which is then used to represent the central tendency for each process with error bar displayed. Detailed values and derivation methods are provided in Table S2. For most emerging low‐carbon steelmaking technologies, direct UPOPs emission data are unavailable due to their early‐stage or conceptual development status. To address this, a mechanism‐based estimation framework, grounded in the physicochemical principles of UPOPs formation, and informed by process‐specific parameters such as operating temperature, oxygen availability, and feedstock composition, was developed (Figure S1 and Text S1). For each emerging route, emission factor ranges were determined by systematically comparing it with a reference conventional process (e.g., BAT BF‐BOF vs. BF‐BOF), incorporating differences of chlorine content in feedstock (Table S6) and scrap input ratio. Details about all steelmaking routes could be seen in Table S3.

Mass balance is a widely adopted approach for estimating material flows in metal production. In this study, mass flows of process inputs are derived based on iron content conservation. Specifically, the mass flow is determined by the iron concentration (percentage of iron) in both the input (C_Fe input_ (%)) and output materials (C_Fe output_ (%)) [74, 75, 76, 77], as well as the process‐specific loss rate (r_loss_ (%)) [77]. Detailed equations are shown below:

(1) $$M_{input}=M_{output}\;*\frac{\frac{C_{Fe\;output}}{1-r_{loss}}}{C_{Fe\;input}}$$

where the M_output_ and M_input_ represent the mass of the input and output materials (t). The C_Fe_ and r_loss_ of emerging technologies could be seen in Figure S4. The emission factors of UPOPs for each production route (EF_route_):

(2) $$E\;F_{route}=\sum \left(EF_{processes}*M_{output}\right)\;$$

This approach enables the estimation of UPOPs emissions across complete steelmaking routes under current and future technological configurations.

### 4.2 Statistical Analysis

Statistical analysis was performed to evaluate the probability distributions of emission factors for EAF(Scrap) and IOS in the log space. Candidate distributions (normal, lognormal, gamma, and Weibull) were fitted by maximum‐likelihood estimation, and goodness‐of‐fit was assessed using the Kolmogorov–Smirnov test (p > 0.05 indicating no significant deviation from the fitted distribution), together with information criteria (lower AIC/BIC) and graphical diagnostics (density, Q‐Q, CDF, and P‐P plots). The error bars and their definitions are provided in the figure legends.

### 4.3 Scenarios

Scenario‐based steel production has been extensively developed by international institutions and research. To ensure consistency and representativeness, we conducted a structured comparison of these major roadmap data (see Tables S7 and S8), covering organization type, modeling approach, scenario design, time horizon, system boundary, technology resolution, economic detail, transparency of assumptions, and explicit climate alignment. The IEA and MPP pathways provide the defensible activity inputs for our UPOPs accounting. They offer internally consistent, quantitative trajectories to 2050 covering steel output, process composition, and key abatement deployment. They are explicitly anchored to climate goals, and provide explicit regional coverage with sufficiently detailed technology differentiation. Technology shifts are also governed by system‐wide cost considerations in IEA and MPP data, providing a comparable baseline for pathway selection. By contrast, several other roadmaps were not used here because they are designed primarily for progress tracking rather than scenario projection, restrict attention to a single lever, or lack the transparency and route‐level detail needed to match the accounting scope of UPOPs. Therefore, steel production data derived IEA and MPP serve as inputs to our emission and health risk models [23, 33].

The Base scenario (aligned with IEA Stated Policies Scenario) reflects the continuation of existing and announced national policy commitments without additional efforts to decarbonize the steel sector. It assumes no new technological mandates, no carbon pricing, and no coordinated industrial transformation. The global steel production could achieve 2.54 Gt in 2050 with 36% from BF‐BOF and 52% from EAF(Scrap). SDS_LTE (aligned with IEA Sustainable Development Scenario) outlines a well‐below‐2°C pathway by 2050. The carbon reduction in this scenario relies on significant material‐efficiency improvements (20% reduction of global steel production in 2050 compared with Base) and broad deployment of relatively mature low‐emissions technologies. The 33%, 38%, 19%, and 10% of total steel is from BF‐BOF‐based routes, EAF(Scarp), DRI‐based routes, and SR‐based routes in 2050. Base_HTE (aligned with MPP Baseline Scenario) represents higher technology expectations than the Base with the same 2050 steel production, in which 43% of total from BF‐BOF‐based routes, 35% from EAF(Scarp), 8% from DRI‐based routes, and 14% from SR‐based routes. It assumes only the natural uptake of low‐emissions technologies when economically viable. TM_HTE (aligned with the MPP Tech Moratorium Scenario) imposes a technology moratorium. A regulatory moratorium takes effect from 2030, banning new investments in conventional high‐emissions technologies. CC_HTE (aligned with the MPP Carbon Cost Scenario) introduces an economy‐wide carbon cost that increases over time and progressively favors low‐emission routes. TM_HTE and CC_HTE represent well‐below‐2°C‐consistent policy stringency with the same global steel production with Base. In 2050, BF‐BOF‐based, DRI‐based, SR based and EAF(Scrap), and other net‐zero steelmaking routes account for 6%, 29%, 15%, 39,% and 11% of total production under TM_HTE, respectively, while the value for CC_HTE are 6%, 41%, 7%, 40%, and 6%. Details of the scenarios are provided in Table S8.

### 4.4 UPOPs Emission Model

Emissions of UPOPs under climate goal were estimated at five‐year intervals from 2025 to 2050. A bottom‐up calculation framework was used, combining technology‐specific production volumes with corresponding emission factors. Steel production volumes and technology shares were adapted from the original report from IEA and MPP reports. Production values between 2019 and 2050 were interpolated based on the expected commercial readiness year of each steelmaking route and a linear ramp‐up in capacity share from that year to the target level in 2050. This approach reflects both the staggered maturity of emerging technologies and plausible diffusion dynamics across the asset base. The global steel production and technology shares under the five scenarios from 2019 to 2050 are shown in Figure S5. Regional distribution of UPOPs emissions under the climate goal was estimated under Base and SDS_LTE. Because the MPP data are derived from a bottom‐up, asset‐level model that does not represent inter‐regional trade or capacity relocation, MPP data is only used for global/process‐level sensitivity analyses of UPOPs emission. Regional emission distributions and health risk estimates based on IEA data to ensure consistent and plausible regional capacity inputs. Regional steel production and technology share are provided in Figure S6.

The UPOPs emissions from a specific steelmaking route (Emission_route_) in a given year were calculated as:

(3) $$\begin{array}{ccc} & & E\;mission_{route}\\ & & =EF_{route}*\mathrm{Production}\;\mathrm{of}\;\mathrm{specific}\;\mathrm{steelmaking}\;\mathrm{route} \end{array}$$

Total of global or regional emissions of specific UPOP (Emission_total_) could be calculated as:

(4) $$E\;mission_{total}=\sum Emission_{route}\;$$

### 4.5 Health Risk Model

Inhalation is the primary pathway of human exposure to atmospheric UPOPs released from steel production. Here, a regional‐scale multimedia steady‐state model was established to simulate UPOPs emissions from industrial sources. This type of model has been widely applied by national environmental agencies (e.g., U.S. EPA [78, 79], ECHA [80], RIVM [81]) and international organizations such as the OECD [79]. In the atmosphere, UPOPs predominantly occur in gaseous and particle‐bound phases. Their removal is mainly governed by gaseous and particle dry deposition and a series of gaseous degradation processes [49, 82, 83, 84]. On this basis, the model simulates steady‐state atmospheric concentrations under source–sink equilibrium. For each UPOP species:

(5) $$F\;=\;\mathrm{Emissio}n_{\mathrm{total}}/\left(A*365*24*3600\right)$$

(6) $$\mathrm{Steady}\;\mathrm{Con}\;._{\mathrm{gas}}=\;F*f_{g}/\left(v_{g}+\frac{\ln2}{\mathrm{halftime}}*H\right)$$

(7) $$\mathrm{Steady}\;\mathrm{Con}\;._{\mathrm{particle}}=\;F*f_{g}/v_{p}$$

(8) $$\mathrm{Steady}\;\mathrm{Con}\;._{\mathrm{atmosphere}}=\;\mathrm{Steady}\;\mathrm{Con}._{\mathrm{gas}}+\mathrm{Steady}\;\mathrm{Con}._{\mathrm{particle}}$$

(9) $$\mathrm{Steady}\;\mathrm{Con}\;._{\mathrm{total}}=\sum \mathrm{Steady}\;\mathrm{Con}._{\mathrm{atmosphere}}\;$$

where Steady Con._gas_, Steady Con._particle_, Steady Con._atmosphere_ and Con._total_ represent the steady‐state concentrations of a given pollutant in the gas phase (µg TEQ·m^−3^), particle phase (µg TEQ·m^−3^), total atmosphere (µg TEQ·m^−3^) and total concentration of all UPOPs (µg TEQ·m^−3^), respectively. *F* and *A* denote the emission flux from steel‐producing regions (µg TEQ·m^−2^·s^−1^) and the surface area of the emission region (m^2^), while *H* represents the atmospheric mixing height (m) [85, 86, 87, 88, 89]. The f_g_, v_g_, v_p_, and halftime correspond to the gas‐particle partitioning coefficient [90, 91, 92, 93, 94], gas‐phase deposition velocity (m·s^−1^) [95, 96], particle‐phase deposition velocity (m·s^−1^) [97], and gas‐phase half‐life (s) [98, 99, 100, 101], respectively. All the parameters are temperature‐adjusted under different climate scenarios (Text S2).

The lifetime inhalation exposure risk for the population (R) is given by:

(10) $$R=Steady\;Con._{total}*Inhalation\;Unit\;Riks_{TEQ}$$

Inhalation Unit Riks_TEQ_ µg^−1^·m^3^ represents the cancer risks continuous exposure to pollutants equal to 1 µg·m^−3^ TEQ. Parameter values are taken from official guidebooks published by OEHHA and the U.S. EPA [48, 49].

For 2050, the expected number of cancer cases in populations living in specific steel‐producing regions was calculated by coupling scenario‐based risk estimates with regional population size and age structure. The equations are as follows:

(11) $$\mathrm{Cancer}\;\mathrm{case}\;s_{\mathrm{age}}=\;R*\mathrm{Populatio}n_{\mathrm{age}}*\mathrm{Life}\;\mathrm{expectanc}y_{\mathrm{age}}$$

(12) $$\mathrm{Life}\;\mathrm{expectanc}y_{\mathrm{age}}=\frac{\int_{\mathrm{age}}^{104}S_{\mathrm{age}}d\left(\mathrm{age}\right)}{S_{\mathrm{age}}}\;$$

(13) $$\mathrm{Cancer}\;\mathrm{case}\;s_{\mathrm{total}}=\sum \mathrm{Cancer}\;\mathrm{case}s_{\mathrm{age}}\;$$

where Cancer cases_age_, Population_age_, Life expectancy_age__e_, and S(age) denote the expected cancer cases, population size, life expectancy, and survival rate for a given age group in a specific region (Dataset S5) [51]. Cancer cases_total_ represents the total expected number of excess cancer cases. Population data for each scenario were obtained from the Wittgenstein Centre's database and aligned with the Base and SDS_LTE climate targets (Data S5). All the parameters used in the model could be seen in Table S9.

### 4.6 Uncertainty Analysis

The modeling framework was constructed in a stepwise manner, including an emission inventory model under climate scenarios, an atmospheric multimedia steady‐state model, and a health risk model. The emission inventory was estimated using the emission factor method, recommended by the European Monitoring and Evaluation Programme/European Environment Agency (EMEP/EEA) [41] and the UNEP [102]. Production data were obtained from IEA and MPP official reports [23, 33], while emission factors were taken from UNEP guidelines and more than 100 field measurements worldwide. The multimedia model incorporated UPOPs atmospheric behavior parameters and meteorological conditions such as mixing layer height. Meteorological parameters were derived from field monitoring data in Northeast and North China [85, 88], Austria [89], and New York [86], whereas UPOPs physicochemical properties were compiled from the literature and official reports. The health risk model adopted inhalation unit risks from national and international agencies, and population projections were obtained from the Wittgenstein Centre [51], which has also been adopted in the IPCC Sixth Assessment Report (AR6) as the baseline population dataset for climate scenario analysis.

The steel‐production scenarios simulated by the IEA and MPP are used as steel production inputs, which are also referenced in authoritative assessments, such as IPCC AR6 mitigation report [103]. But no single set of scenarios can realistically span the full space of possible futures. Further modeling efforts are needed to broaden the space of plausible futures and to more comprehensively test the robustness of projections against this structural uncertainty.

Additionally, field measurements of emission factors for the steel sector remain limited. Consistent with widely used UPOPs inventory approaches, including the UNEP Toolkit and the EMEP/EEA air pollutant emission inventory guidebook 2023, the same set of emission factors and uncertainty ranges are applied across regions. To quantify emission uncertainty, probability distributions of emission factors were assigned based on available field measurements (Figures S2 and S3), and their variability ranges were propagated through the emission models as shown in Figure 1. Uncertainty bounds of estimating results (Data S1–S4) are calculated by the lower and upper limits of emission factors. From a route perspective, emission factors for EAF(Scrap) (0.522–3.666 µg TEQ·t^−1^ for PCDD/Fs, the dominant emitted UPOPs) and BF‐BOF‐based routes (0.342–3.982) µg TEQ·t^−1^ exhibit relatively large uncertainty (Figure 1), driven primarily by the EAF(Scrap) and IOS stages. The variations of global UPOPs emissions under Base, SDS_LTE, Base_HTE, TM_HTE, and CC_HTE are 1319–3462 g TEQ, 957–7172 g TEQ, 1156–8788 g TEQ, 930–5190 g TEQ, and 967–5301 g TEQ, respectively. At the regional level, under the Base scenario, the values are 495–4405 g TEQ for China, 205–1757 g TEQ for India, 40–286 g TEQ for the US, 88–752 g TEQ for the EU, 29–115 g TEQ for the Middle East, 88–816 g TEQ for Central and South America, and 33–251 g TEQ for Africa, which show the similar relative deviation comparable to those under SDS_LTE. The uncertainty is mainly contributed by emission factors of EAF(Scrap) and IOS stages. Sensitivity is higher in BF‐BOF and EAF(Scrap) ‐dominated settings, such as the Base, SDS_LTE, and Base_HTE, and regions with high BF‐BOF shares. Nevertheless, the main qualitative conclusions including the emission trends, regional distribution, and emission composition of steelmaking routes, remain unchanged. However, further research is needed to expand the emission‐factor database and develop region‐specific UPOPs emission factors to strengthen the robustness of future assessments. Continued atmospheric monitoring and source apportionment will also be essential for validating model projections and predictive accuracy.

For the health risk model, we quantified parameter‐driven variations in regional lifetime inhalation cancer risks under the Base and SDS_LTE scenarios (Data S6) and associated cancer cases. Under the SDS_LTE, the variations of cancer cases caused by lifetime inhalation are 670–5368 for China, 968–6443 for India, 22–130 for the United States, 181–1394 for the EU, 23–96 for the Middle East, 23–184 for Central and South America, and 31–229 for Africa. Under Base, the corresponding ranges are 918–8318, 1416–12326, 18–199, 246–2133, 25–99, 39–365 and 40–310, respectively. Despite the uncertainty in absolute values, the spatial pattern and ranking of regional risks remain robust. The overall uncertainty is mainly driven by uncertainty in half‐life time of UPOPs, particularly for higher‐emitting PCDD/Fs, and in the particle deposition velocity. Reducing these uncertainties will require more field measurements and laboratory simulations to better constrain key processes. More evidence on regional differences in these parameters is needed to support higher‐resolution simulations in future work.

In terms of model boundaries, this study focuses on primary emissions from steelmaking sources to the environment and the associated human inhalation risks. With atmospheric warming, re‐emissions from legacy reservoirs may also contribute to the atmospheric burden. The partitioning and exchange of UPOPs among different environmental media could be different, and the associated exposure from other ways, such as food intake [104], should be explored in further research. Rigorously quantifying such secondary releases, environmental behaviors, and associated exposures, would require a larger coupled multimedia framework and stronger empirical constraints. Future work should therefore incorporate secondary sources and quantify their incremental contributions to emissions and population risks under climate scenarios. In addition, UPOPs across different phases could cause more complex health end‐points. More toxicological evidence and quantitative results could largely support future risk modeling, which is still challenging and limited.

## Author Contributions

Yuxiang Sun collected and analysed the data, and authored the paper; Qiuting Yang, Jianghui Yun, Yujue Yang, and Junhao Tang collected and analysed the data; Qian Liu and Minghui Zheng analysed the data and revised the paper. Guorui Liu conceptualized the study, revised the paper, and acquired funding.

## Funding

The Strategic Priority Research Program of the Chinese Academy of Sciences (grant numbers XDB0750400) (G.L.); the financial support from Zhejiang Normal University (YS304025934)

## Conflicts of Interest

The authors declare no conflicts of interest.

## Supporting information




**Supporting File 1**: advs74797‐sup‐0001‐SuppMat.docx.

**Supporting File 2**: advs74797‐sup‐0002‐DataFile.zip.

## Data Availability

The data that support the findings of this study are available in the supplementary material of this article.
