# Supplementary material for: Climate Action Impacts on Steelmaking Emissions of Persistent Organic Pollutants Highlight a Gap Between the Paris Agreement and the Stockholm Convention
Source: Adv Sci (Weinh). 2026 Mar 25;13(29):e19769. doi: 10.1002/advs.202519769 (PMC13205816; doi:10.1002/advs.202519769)
Supplement: Supplementary file 1 — Supporting File 1: advs74797‐sup‐0001‐SuppMat.docx. [file ADVS-13-e19769-s001.docx]

Supporting Information

**Climate Action Impacts on Steelmaking Emissions of Persistent Organic Pollutants Highlight a Gap Between the Paris Agreement and the Stockholm Convention**

*Yuxiang Sun, Qiuting Yang, Jianghui Yun, Yujue Yang, Junhao Tang, Qian Liu, Minghui Zheng, Guorui Liu**

Supporting Information Text 1

**Qualification and Quantification of UPOPs formation and emission from emerging steelmaking routes**

Previous studies have established that PCDD/Fs can be formed through two main mechanisms: precursor pathways and de novo synthesis. The precursor mechanism involves the transformation of structurally related compounds, such as chlorinated phenols and benzenes, into PCDD/Fs via condensation reactions^[1]^. In steelmaking, potential precursor-based emissions may occur during scrap pre-heating in EAF(Scrap), where volatilization of residual organic materials could lead to PCDD/Fs release and formation^[2]^. However, in most high-temperature metallurgical operations (e.g., blast furnaces and converters), both PCDD/Fs and their precursors are largely decomposed due to thermal conditions, making precursor-derived or direct emissions unlikely^[3, 4]^. In contrast, de novo synthesis is widely regarded as the dominant pathway for unintentional PCDD/F formation in steelmaking processes^[5, 6, 7, 8, 9, 10, 11]^. It involves the formation of PCDD/Fs from chemically unrelated carbon matrix in the presence of chlorine donors, molecular oxygen, and transition metal catalysts (Cu, Fe) within a temperature range of 200-450°C^[12]^. This mechanism is especially relevant in flue gas cooling zones, such as those downstream of BF, BOF, or in the lower sections of sinter beds, where conditions favor de novo activity^[3, 4]^. These processes are well-documented in UNEP technical guidance documents and peer-reviewed studies. Studies also suggests that PCBs and PCNs may share similar de novo pathways^[13]^, and that PeCBz and HCB concentrations correlate with PCDD/F formation levels^[14, 15]^.

To assess the potential for UPOPs formation in emerging steelmaking technologies, we first evaluated whether the process configurations meet the key physicochemical conditions required for de novo synthesis. The main influencing factors include: Chlorine donors (organic or inorganic) ^[14, 15, 16, 17]^, oxygen^[18]^, Carbon matrices^[12]^ (e.g., soot, fly ash, residual organics), temperature window (200–450°C)^[3]^ and catalysts (primarily Cu and Fe oxides) ^[19, 20, 21]^. Among these, temperature is the threshold determinant. Copper and iron oxides are effective catalysts that facilitate the chlorination and oxidative coupling of carbon structures, while chlorine serves as the essential halogen source for all UPOPs.

Following the qualitative assessment, we employed chlorine content as a surrogate variable for preliminary quantification of PCDD/F emissions. A growing body of research supports the use of chlorine concentration as a quantitative predictor for PCDD/Fs formed via de novo synthesis. Stanmore (2002)^[17]^ proposed a theoretical model showing a linear dependence of PCDD/F formation rate on HCl partial pressure. Watanabe et al. (2007)^[14]^ reported a significant linear correlation (r = 0.81) between low-volatile organic chlorine and total PCDD/F concentrations in municipal solid waste incinerators. Jiang et al. (2023)^[15]^ observed a near-linear increase in PCDD/F emissions with chlorine content ranging from 2.91% to 8.24%, where total PCDD/F concentrations rose from 2.71 to 8.25 ng/Nm³, and I-TEQ from 0.029 to 0.113 ng TEQ/Nm³. Thomas and McCreight (2008)^[16]^ statistically analyzed 17 combustion categories and established a log–log linear regression between chlorine content (ppm) and EFs (ng I-TEQ/kg): log(EF) = (0.4 ± 0.2) × log(Cl) − (1 ± 0.8). Despite the statistical collinearity between copper and chlorine (R² = 0.75), their analysis confirmed a consistent amplification effect of chlorine content on PCDD/F emissions. Taken together, these findings support the use of chlorine content as a valid and effective predictor of PCDD/F emission intensity under de novo conditions. Accordingly, we estimated ranges of EFs for emerging technologies by scaling from the baseline process values (see Table S2 and Fig. S2), with chlorine content in raw materials serving as the principal adjustment factor. For the emission model, we adopted the midpoint between the baseline value and the upper bound as the representative EF, while the full range was retained for sensitivity analysis.

Supporting Information Text 2

**Warming perturbation of the health risk model**

Different warming levels across climate scenarios can lead to differences in the atmospheric behavior of UPOPs. We therefore considered the impacts of scenario-specific temperature increases on the model parameters relevant to atmospheric processes.

**Atmospheric mixing height**

Available evidence suggests that the mixing height response to mid-century warming is generally positive but weak. Chen et al. reported that under a 2050s climate scenario where the annual-mean daily maximum temperature increases by 1.3 °C, the annual-mean mixing height increases by only 2%^[22]^. In addition, Hu et al. ^[23]^reported, despite national-mean winter warming of about 0.7-1.0 °C, the national-mean mixing height in winter increase is only 2.1 m to 15.1 m, supporting a meter-to-tens-of-meters weak response. Taken together, although mixing height and warming are often positively correlated, under the mid-century conditions considered here (warming of roughly 1.5-2 °C), changes in mixing height are expected to be present but limited. Accordingly, in our analysis we applied a 2% (1.5 °C perturbation) adjustment for well-below-2°C scenarios (SDS_LTE, TM_HTE, CC_HTE) and 3% adjustment (2 °C perturbation) for Base scenarios (Base, Base_HTE) to the baseline mixing height to represent the warming perturbation.

**Particle-gas partitioning coefficient (f_p_)**

Particle-gas partitioning influences the environmental fate of UPOPs. Warming tends to shift semi-volatile UPOPs from the particle phase to the gas phase. Harner et al. reported that for UPOPs such as PCDD/Fs and PCBs, the temperature dependence of octanol–air partition coefficient (K_OA_) over 0-50 °C can be approximated by an Arrhenius-type relationship^[24]^:

$Log_{10}K_{OA}=A+B/T$ (1)

where A and B are compound-specific constants, with B = 3200-5541 (K) for PCDD/Fs and PCBs. Therefore, a temperature perturbation ΔT leads to:

$\Delta Log_{10}K_{OA}=-(B*\Delta T)/T^{2}$ (2)

In the absorption-based gas–particle partitioning frameworks (e.g., the Pankow absorption model and the widely used Harner–Bidleman formulation), particle-gas partition coefficient (K_p_) increases with K_OA_, and particle-bound fraction (f_p_) is increased with K_OA_^[25, 26]^, which could be expressed by following equation:

$K_{p}\propto K_{OA}$ (3)

$f_{p}'/{(1-f}_{p}')=\frac{K_{p}'}{K_{p}}*f_{p}/{(1-f}_{p})$ (4)

Taking a representative temperature of T = 298 K and ΔT = 1.5 K and 2 K in 2050 under climate scenarios:

$\frac{K_{p}^{'} 1.5K}{K_{p}}=0.81-0.88$ (5)

$\frac{K_{p}^{'} 2 K}{K_{p}}=0.75-0.85$ (6)

According the equation (1-6), the original and temperature-adjusted parameters are summarized in Table S9.

**Gas-phase dry deposition velocity**

Dry deposition is an important removal pathway for atmospheric UPOPs. In the full resistance framework, gas-phase dry deposition for gaseous chemicals is expressed as:

$V_{g}=1/(R_{a}+R_{b}+R_{c})$ (7)

Where R_a_, R_b_ and R_c_ are the aerodynamic resistance, the quasi-laminar (boundary-layer) resistance and the surface (canopy) resistance^[27, 28]^. However, for UPOPs, R_c_ is not parameterized and is often ignored in atmospheric UPOP models. Thus, the gas-phase dry deposition velocity of UPOPs is represented as:

$V_{g}=1/(R_{a}+R_{b})$ (8)

Although R_a_ and R_b_ co-vary with atmospheric stability and radiation on a daily basis, translating such co-variations into a deterministic temperature dependence is not currently supported for UPOPs. The direct temperature sensitivity in this framework mainly enters through R_b_, which can be expressed as:

$R_{b}=2({S_{c}/P_{r})}^{\frac{2}{3}}/(k*u)$ (9)

Here, k is the von Kármán constant, u is the friction velocity, P_r_ is the Prandtl number (approximately = 0.7 for air) and S_c_ is the Schmidt number:

$S_{C}=v/D$ (10)

where $\nu$is the kinematic viscosity and D is the molecular diffusivity. Over the small warming perturbations considered here, the temperature dependence of S_c_ is weak. Under a Sutherland-type dependence, S_c_ exhibits a dependence of the form T/(T+S), where S is the Sutherland constant (110 K for air). Therefore, taking a representative temperature of T = 298 K and ΔT = 1.5 K and 2 K in 2050 under climate scenarios, the warming leads to only a sub-percent change in S_c_ (on the order of 10^-3^), and consequently the induced change in R_b_ and V_g_ is negligible, at roughly the 0.1% level.

Accordingly, the gas-phase dry deposition velocity was not treated as a temperature-dependent parameter under warming perturbations in our simulations.

**Particle-phase deposition velocity**

UPOPs are predominantly associated with fine aerosols, for which gravitational settling is not an appropriate representation. Consistent with the resistance-based treatment above, the deposition velocity (V_p_) is approximated as $V_{p}=1/(R_{a}+R_{b})$. R_a_ and R_b_ are defined consistently with the gas-phase formulation within the same resistance framework. As discussed above, the Particle-phase deposition velocity was not treated as a temperature-dependent parameter under warming perturbations in our simulations.**​**

**Half-life time of UPOPs**

For the atmospheric reactions of UPOPs, the temperature sensitivity is generally reported to be weak. Brubaker and Hites^[31]^ performed Arrhenius regressions over 306-405 K and explicitly concluded that the temperature dependencies are slight to nonexistent for the studied reactions. Yan et al.^[32]^ developed LFER/PLS models and got the similar conclusion. In addition, Taylor et al. ^[33]^ reported Arrhenius parameters for the reactions of dibenzodioxin and several chlorinated dioxins with OH, noting that multiple reaction channels exhibit negative activation energies. Such negative activation energies imply that the overall temperature dependence can be weak within typical atmospheric temperature ranges.

Accordingly, the atmospheric half-life of UPOPs was not treated as a temperature-dependent parameter under warming perturbations in our simulations.**​**

Figures

Fig. S1. Mechanism-based and process-parameter-grounded^[34, 35]^ estimation framework for Emission factors (EFs) for unintentionally produced persistent organic pollutants (UPOPs) of emerging steelmaking routes. (a) Overview of emerging steelmaking processes and routes; (b) Estimation framework incorporating UPOPs formation mechanisms and process-specific parameters for EF prediction.


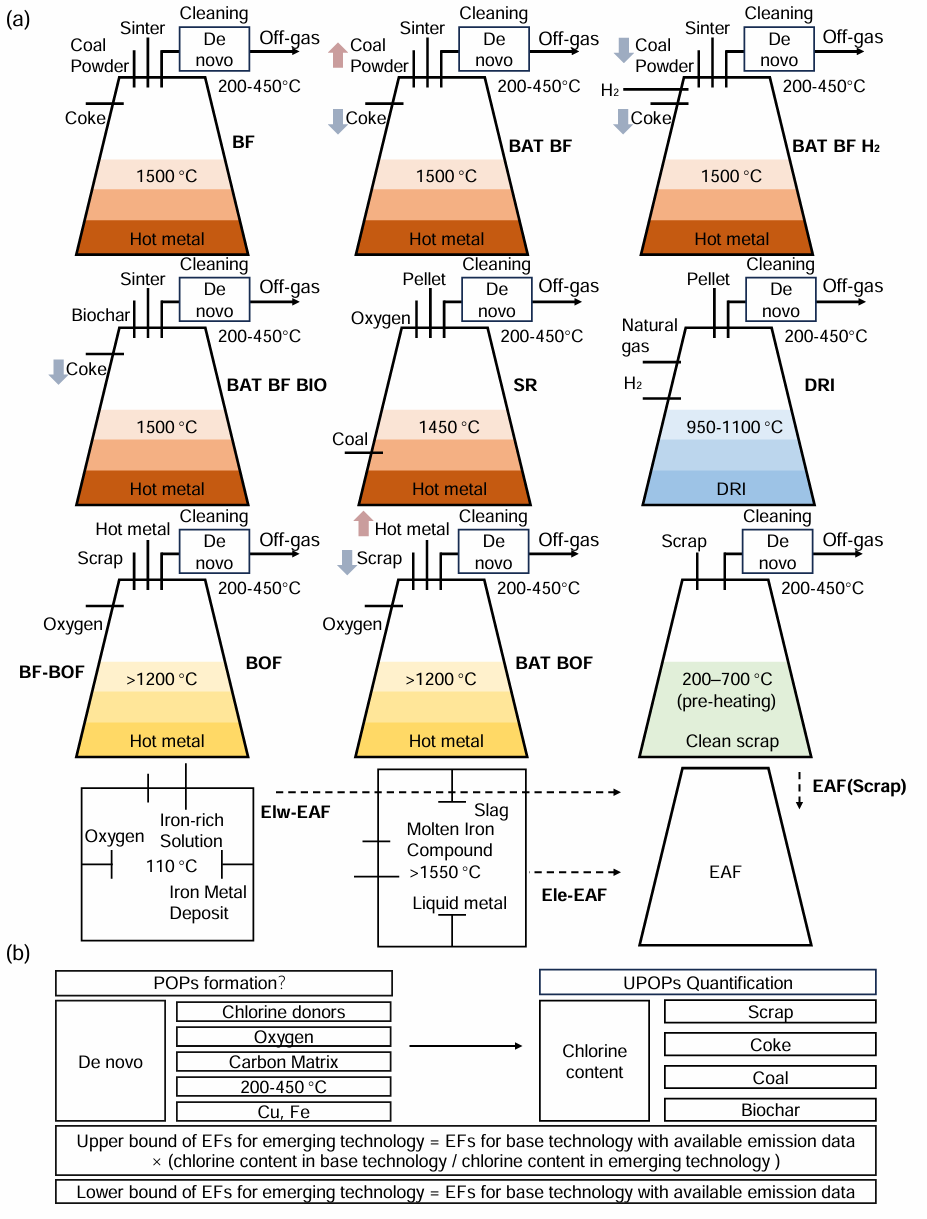


Fig. S2. Statistical analysis of reported Emission factors (EFs) of scrap-based electric arc furnace steelmaking (EAF(Scrap).


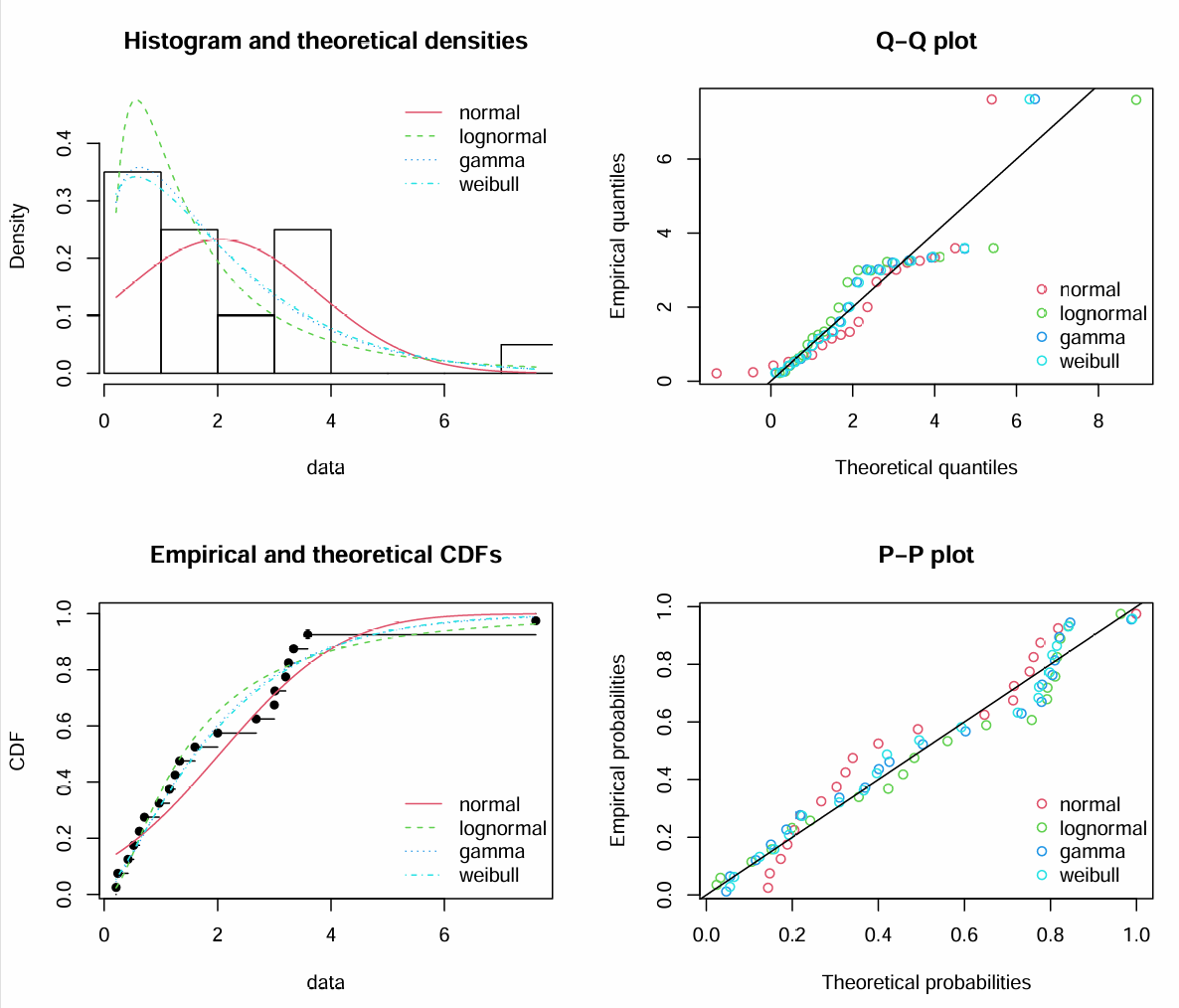


Fig. S3. Statistical analysis of reported Emission factors (EFs) of Iron ore sintering (IOS)


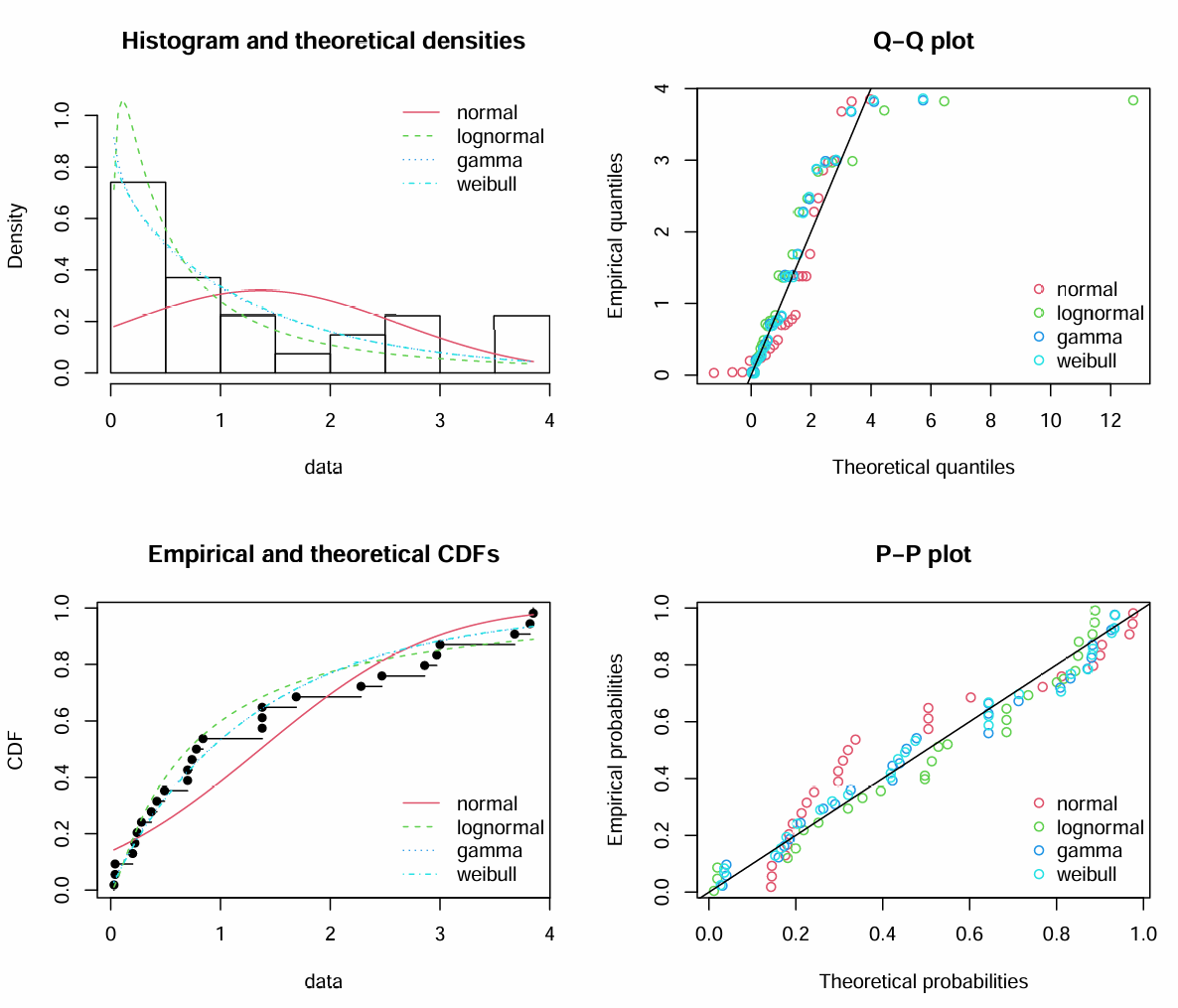


Fig. S4. Process and mass flow of different steelmaking routes^[35, 36, 37, 38, 39, 40, 41]^

(IOS: iron ore sintering; COP: coke production; PLP: iron ore pelletizing; BF: blast furnace ironmaking; BAT BF: BF with best available technology; BAT BF H_2_: BAT BF with hydrogen injection; BAT BF BIO: BAT BF with biomass-based reductant substitution; DRI: direct reduced iron; DRI 100% H_2_: DRI using 100% hydrogen as reductant; DRI 50% H_2_: DRI with 50% hydrogen as reductant; SR: smelting reduction ironmaking; BOF: basic oxygen furnace steelmaking; BAT BOF: BOF with best available technology; BOF (DRI): BOF fed by reduced iron; EAF: Electric Arc Furnace steelmaking)


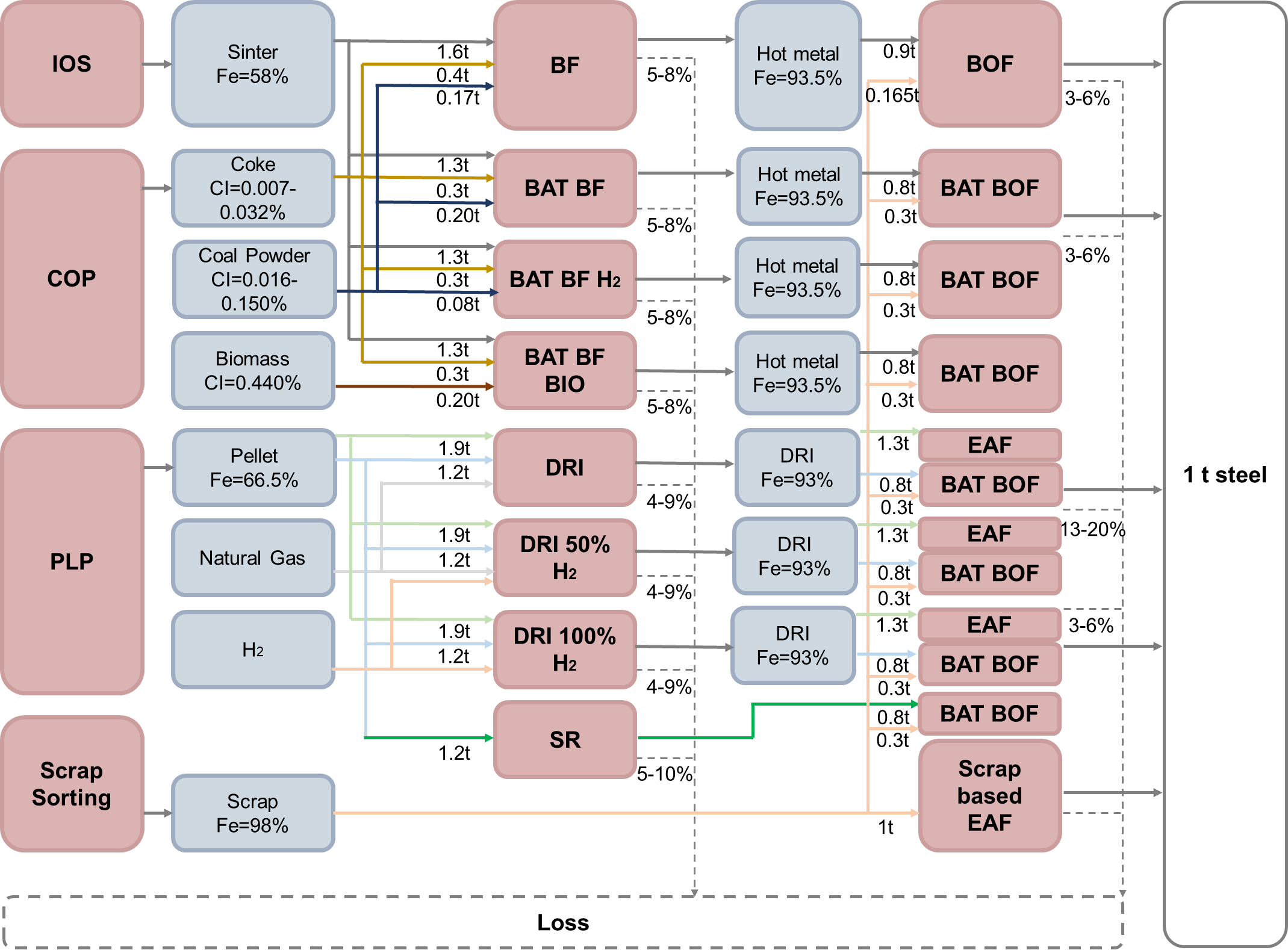


Fig. S5. Global steel production under (a) Base, (b) SDS_LTE, (c) Base_HTE, (d) TM_HTE and (e) CC_HTE (BF-BOF: blast furnace ironmaking-basic oxygen furnace steelmaking route; EAF: scrap-based electric arc furnace steelmaking route; DRI-EAF: Direct-reduced-iron-fed EAF route; SR-BOF: Smelting reduction ironmaking-BOF route; DRI-EAF H₂: DRI-EAF route using hydrogen as the reductant; DRI-EAF CCS: DRI-EAF route with CCS; BAT BF-BOF H₂: BAT BF-BOF route with hydrogen injection; DRI-BOF: DRI-fed BOF route; SR-BOF CCS: SR-BOF route with carbon capture and storage; BAT BF-BOF: BF-BOF route with best available technology; BAT BF-BOF CCS: BAT BF-BOF route with CCS; BAT BF-BOF BIO: BAT BF-BOF route with biomass-based reductant substitution; Ele+Elw-EAF: EAF fed by electrolytic or electrowon iron)


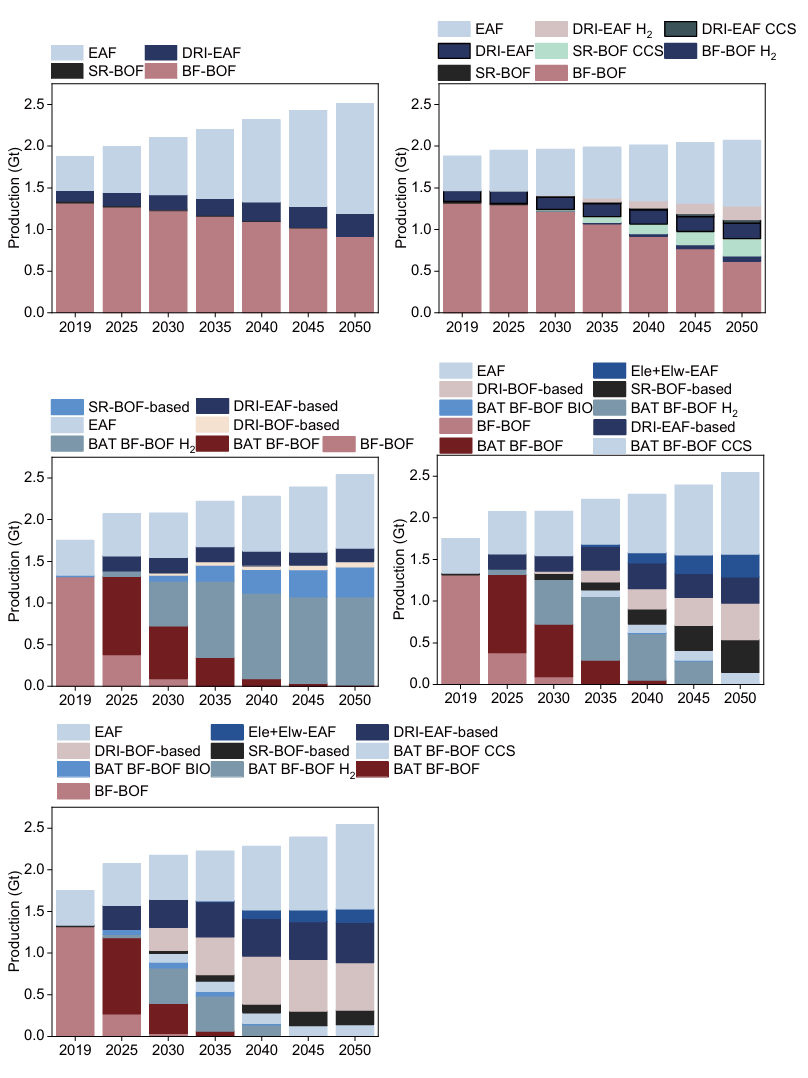


Fig. S6. Regional steel production from each steelmaking routes in 2019 and 2050 under Base and SDS_LTE (BF-BOF: blast furnace ironmaking-basic oxygen furnace steelmaking route; EAF(Scrap): scrap-based electric arc furnace steelmaking route; DRI-EAF: Direct-reduced-iron-fed EAF route; SR-BOF: Smelting reduction ironmaking-BOF route; DRI-EAF H₂: DRI-EAF route using hydrogen as the reductant; DRI-EAF CCS: DRI-EAF route with CCS; BAT BF-BOF H₂: BAT BF-BOF route with hydrogen injection; SR-BOF CCS: SR-BOF route with carbon capture and storage)


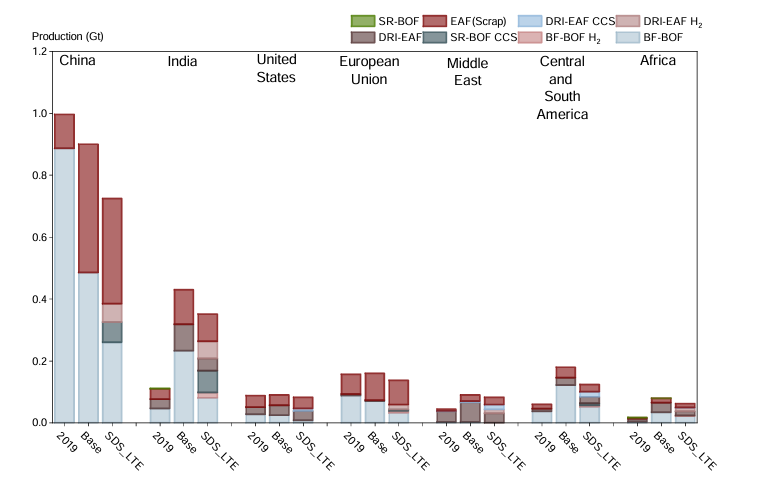


Tables

Table S1. EFs reported for UPOPs

| References | Year | Plant Number | Countries/Regions | EFs for six UPOPs | | | | | |
| --- | --- | --- | --- | --- | --- | --- | --- | --- | --- |
|  |  |  |  | PCDD/Fs | PCBs | HCB | PeCBz | PCNs | HCBD |
| ^[42]^ | 2023 | IOS-1 | China | 0.2 | - | - | - | - | - |
| ^[43]^ | 2017 | IOS-2 | China | 0.7 | - | - | - | - | - |
|  |  | IOS-3 | China | 0.22 | - | - | - | - | - |
| ^[44]^ | 2023 | IOS-4 | China | 1.38 | - | - | - | - | - |
| ^[45]^ | 2022 | IOS-5 | China | 1.38 | - | - | - | - | - |
| ^[46]^ | 2022 | IOS-6 | China | 1.38 | - | - | - | - | - |
| ^[47]^ | 2016 | IOS-7 | Taiwan, China | 1.69 | - | - | - | - | - |
| ^[5]^ | 2014 | IOS-8 | the United Kingdom | 2.47 | - | - | - | - | - |
|  |  | IOS-9 | the United Kingdom | 2.86 | - | - | - | - | - |
| ^[48]^ | 2014 | IOS-10 | Italy | 0.7 | 0.076 | - | - | - | - |
| ^[46]^ | 2012 | IOS-11 | China | 3.68 | 0.38 | 156 | 1079 | - | - |
|  |  | IOS-12 | China | 3.82 | 0.37 | 363 | 1362 | - | - |
|  |  | IOS-13 | China | 2.97 | 0.33 | 684 | 1008 | - | - |
|  |  | IOS-14 | China | 3.85 | 0.38 | 294 | 1217 | - | - |
| ^[49]^ | 2012 | IOS-15 | China | 0.78 | - | - | - | - | - |
|  |  | IOS-16 | China | 0.84 | - | - | - | - | - |
| ^[50]^ | 2019 | IOS-17 | China | 0.28 | - | - | - | - | - |
| ^[7]^ | 2017 | IOS-18 | China | 0.74 | 0.030 | - | - | 0.016 | - |
|  |  | IOS-19 | China | 0.03 | 0.001 | - | - | 0.025 | - |
|  |  | IOS-20 | China | 0.24 | 0.014 | - | - | 0.017 | - |
|  |  | IOS-21 | China | 0.04 | 0.002 | - | - | 0.009 | - |
|  |  | IOS-22 | China | 0.04 | 0.002 | - | - | 0.003 | - |
|  |  | IOS-23 | China | 0.42 | 0.020 | - | - | 0.012 | - |
| ^[51]^ | 2010 | IOS-24 | China | 2.28 | - | - | - | - | - |
|  |  | IOS-25 | China | 0.49 | - | - | - | - | - |
|  |  | IOS-26 | China | 0.37 | - | - | - | - | - |
| ^[52]^ | 2012 | IOS-27 | China | - | - | - | - | 0.002 | - |
|  |  | IOS-28 | China | - | - | - | - | 0.0007 | - |
|  |  | IOS-29 | China | - | - | - | - | 0.001 | - |
|  |  | IOS-30 | China | - | - | - | - | 0.0017 | - |
|  |  | IOS-31 | China | - | - | - | - | 0.0042 | - |
|  |  | IOS-32 | China | - | - | - | - | 0.002 | - |
|  |  | IOS-33 | China | - | - | - | - | 0.002 | - |
|  |  | IOS-34 | China | - | - | - | - | 0.0005 | - |
| ^[53]^ | 2024 | IOS-35 | China | - | - | - | - | - | 1.1 |
|  |  | IOS-36 | China | - | - | - | - | - | 0.1 |
|  |  | IOS-37 | China | - | - | - | - | - | 0.9 |
|  |  | IOS-38 | China | - | - | - | - | - | 0.8 |
| ^[54]^ | 2024 | Regional Investigation in EU (multinational coverage) | EU | 3.00 | - | - | - | - | - |
| ^[2]^ | 2020 | EAF-1 | China | 0.24 | - | - | - | - | - |
| ^[55]^ | 2011 | EAF-2 | Taiwan, China | 0.42 | - | - | - | - | - |
| ^[56]^ | 2019 | EAF-3 | Vietnam | 0.71 | - | - | - | - | - |
|  |  | EAF-4 | Vietnam | 0.21 | - | - | - | - | - |
|  |  | EAF-5 | Vietnam | 3.25 | - | - | - | - | - |
|  |  | EAF-6 | Vietnam | 1.25 | - | - | - | - | - |
| ^[57]^ | 2012 | EAF-7 | Portugal | 3.01 | 0.659 | - | - | - | - |
|  |  | EAF-8 | Portugal | 3.34 | 0.597 | - | - | - | - |
| ^[58]^ | 2007 | EAF-9 | Poland | 0.62 | - | - | - | - | - |
| ^[59]^ | 2002 | EAF-10 | Italy | 1.15 | - | - | - | - | - |
| ^[11]^ | 2005 | EAF-11 | Taiwan, China | 1.6 | - | - | - | - | - |
|  |  | EAF-12 | Taiwan, China | 2 | - | - | - | - | - |
|  |  | EAF-13 | Taiwan, China | 0.52 | - | - | - | - | - |
|  |  | EAF-14 | Taiwan, China | 3.2 | - | - | - | - | - |
| ^[60]^ | 2009 | EAF-15 | Taiwan, China | 3.59 | - | - | - | - | - |
|  |  | EAF-16 | Taiwan, China | 3 | - | - | - | - | - |
|  |  | EAF-17 | Taiwan, China | 2.68 | - | - | - | - | - |
|  |  | EAF-18 | Taiwan, China | 1.33 | - | - | - | - | - |
|  |  | EAF-19 | Taiwan, China | 7.61 | - | - | - | - | - |
| ^[61]^ | 2013 | EAF-20 | China | - | 0.087 | - | - | - | - |
|  |  | EAF-21 | China | - | 0.062 | - | - | - | - |
|  |  | EAF-22 | China | - | 0.073 | - | - | - | - |
| ^[10]^ | 2012 | EAF-23 | China | - | - | 3200 | - | 0.022 | - |
|  |  | EAF-24 | China | - | - | 940 | - | 0.03 | - |
| ^[53]^ | 2024 | EAF-25 | China | - | - | - | - | - | 2.94 |
|  |  | EAF-26 | China | - | - | - | - | - | 46.41 |
|  |  | EAF-27 | China | - | - | - | - | - | 1.89 |
|  |  | EAF-28 | China | - | - | - | - | - | 0.03 |
|  |  | EAF-29 | China | - | - | - | - | - | 0.16 |
|  |  | EAF-30 | China | - | - | - | - | - | 0.10 |
|  |  | EAF-31 | China | - | - | - | - | - | 1.17 |
|  |  | EAF-32 | China | - | - | - | - | - | 0.12 |
|  |  | EAF-33 | China | - | - | - | - | - | 1.28 |
|  |  | EAF-34 | China | - | - | - | - | - | 0.56 |
|  |  | EAF-35 | China | - | - | - | - | - | 0.02 |
|  |  | EAF-36 | China | - | - | - | - | - | 0.07 |
| ^[62]^ | 2011 | National Invetiagtion (>30 Plants) | Japan | 0.97 | - | 1900 | 1200 | - | - |

**Table S2.** EFs of IOS, COP, BF, BOF, Pellet and EAF(Scrap) for scenario-based emission model

| Current Steelmaking Processes | EFs (μg/t HCB and PeCBz, μg TEQ/t for the others) | | | | | |
| --- | --- | --- | --- | --- | --- | --- |
|  | PCDD/Fs | PCBs | HCB | PeCBz | PCNs | HCBD |
| IOS | 0.172-2.91(0.708)^c^ | 0.003-0.324(0.033)^c^ | 177-600(327)^c^ | 1015-1324(1159)^c^ | 0.001-0.012(0.004)^c^ | 0.173-1.625(0.531)^c^ |
| COP | 0.03^a^ | 0.002^a^ | 1^a^ | 1^a^ | 0.001^a^ | 1.5^b^ |
| BF | 0.01^a^ | 0.001^a^ | 1^a^ | 0^d^ | 0^d^ | 0^d^ |
| BOF | 0.1^a^ | 0.001^a^ | 2^a^ | 0^d^ | 0.001^a^ | 0^d^ |
| Pellet | 0.12^b^ | 0^d^ | 30^b^ | 0^d^ | 0^d^ | 0^d^ |
| EAF(Scrap) | 0.522-3.666(1.384)^c^ | 0.053-0.564(0.173)^c^ | 2000^a^ | 1200^a^ | 0.020-0.031(0.026)^c^ | 0.043-3.717(0.401)^c^ |

a: data from UNEP Toolkit; b: data from EMEP/EEA air pollutant emission inventory; c: geomean of reported data calculated in log space with the log-normal one-standard-deviation range and back-transformed to the original scale; d: not considered as an emission source

**Table S3.** Currently used and emerging low-carbon steelmaking routes^[34, 35]^

| Steelmaking Routes | Base Routes | Abbreviation | Process Description | Mass Flow | Year of commercial availability |
| --- | --- | --- | --- | --- | --- |
| A conventional sinter-based blast furnace–basic oxygen furnace route using commercial operating parameters. | - | BF-BOF | Iron ore sinter and coke (450 kg per ton of pig iron) are charged into the blast furnace, combined with pulverized coal injection (PCI, 195 kg per ton of pig iron), facilitating the reduction reactions to produce pig iron. The pig iron is subsequently transferred to a basic oxygen furnace (BOF), where it is combined with a certain proportion of scrap (16.5%) and undergoes oxygen blowing for decarburization, resulting in the production of steel. | 1t steel=0.9t pig rion+0.165t scrap=405 kg coke+1.6t sinter +170 kg PCI | 2020 |
| A typical sinter-based blast furnace–basic oxygen furnace route reflecting industry-average energy and emission performance. | - | BF-BOF | The current industry average is represented by the commercial IOS+COP-BF-BOF route. | 1t steel=0.9t pig rion+0.165t scrap=405 kg coke+1.6t sinter +170 kg PCI | 2020 |
| A sinter-based blast furnace–basic oxygen furnace configuration incorporating best available technologies to minimize emissions. | commercial IOS+COP-BF-BOF | BAT BF-BOF | By applying the best available technologies (BAT), such as enhanced gas recovery and oxygen-enriched blast, energy efficiency and scrap utilization rates are further improved. Measures including an increased pulverized coal injection (PCI) ratio (230 kg per ton of hot metal), a higher scrap ratio (30%), an overall heating efficiency gain (10%), and the adoption of top gas recycling enable a 15% reduction in the coke input to the blast furnace. | 1t steel=0.8t pig rion+0.3t scrap = 344 kg coke+1.3t sinter +196 kg PCI | 2020 |
| A BAT-level blast furnace–basic oxygen furnace system utilizing hydrogen as a partial replacement for pulverized coal injection. | commercial IOS+COP-BF-BOF | BAT BF-BOF H2 | Building on the BAT process, part of the pulverized coal injection (PCI) is replaced with green hydrogen to further reduce fossil carbon emissions. Specifically, hydrogen substitutes for 120 kg of coal per ton of hot metal, out of a total PCI rate of 230 kg per ton of hot metal. | 1t steel=0.8t pig rion+0.3t scrap = 344 kg coke+1.3t sinter +88 kg PCI +H2 | 2025 |
| A BAT-level blast furnace–basic oxygen furnace system utilizing biochar as replacement for pulverized coal injection. | commercial IOS+COP-BF-BOF | BAT BF-BOF BIO | In the BAT process, renewable biomass (e.g., charcoal) is used to substitute pulverized coal injection (PCI) into the blast furnace, thereby reducing net carbon emissions. | 1t steel=0.8t pig rion+0.3t scrap = 344 kg coke+1.3t sinter +196 kg biochar PCI | 2020 |
| A future-oriented blast furnace–basic oxygen furnace system integrating advanced sintering, carbon capture, or hydrogen enrichment technologies. | commercial IOS+COP-BF-BOF | BAT BF-BOF H_2_ | An innovative blast furnace–basic oxygen furnace (BF–BOF) route builds upon the conventional BAT process by integrating advanced measures such as hydrogen injection and extreme top gas recycling. This configuration corresponds to the BAT IOS+COP-BF-BOFF H2 PCI route. | 1t steel=0.8t pig rion+0.3t scrap = 344 kg coke+1.3t sinter +88 kg PCI +H2 | 2025 |
| An electric arc furnace route primarily using recycled scrap steel with minimal direct carbon emissions. | - | EAF(Scrap) | A pre-treatment (preheating) step is often employed, particularly for scrap contaminated with impurities such as oil, grease, or coatings. In the electric arc furnace (EAF) process, high-temperature electric arcs are used to heat and melt the scrap, producing molten steel. | 1t sorted scrap = 1t steel (fully recycled as stated by the World Steel Association) | 2020 |
| A hybrid route combining electrowinning of iron from ore followed by melting in an electric arc furnace. | - | Elw-EAF | Direct iron ore electrolysis process in which iron ore particles are suspended in aqueous alkaline solution in 110°C. Current passing through the solution breaks down ore into oxygen and iron, which crystallises on cathode. Iron is fed into EAF where small amount of metallurgical coal (or pre-treated biomass) is added to supply carbon required to turn iron into steel. | - | 2035 |
| An emerging pathway that uses high-temperature electrolysis to produce iron fed into EAF for steelmaking. | - | Ele-EAF | Molten Ore Electrolysis process in which iron is made via direct electrolysis of molten iron ore or a high-temperature (>1550°C) solution of it, similar to today’s aluminium smelting. Molten iron is fed into EAF and a small amount of metallurgical coal (or pre-treated biomass) is added to supply carbon required to turn iron into steel. | - | 2035 |
| A direct reduced iron-electric arc furnace route using pelletized iron ore and natural gas as the reductant. | - | DRI-EAF | Pelletized iron ore is reduced to DRI in a shaft furnace, where natural gas, reformed into a mixture of CO and H_2_, serves as the reductant, replacing coal typically used in blast furnace routes. The resulting DRI is then charged into an electric arc furnace (EAF), where it is melted to produce molten steel. | 1t steel= 1.3t DRI = 1.9t Pellet | 2020 |
| A direct reduced iron-electric arc furnace pathway equipped with carbon capture and storage (CCS) to reduce process emissions. | pellet-DRI-EAF | DRI-EAF CCS | Pellet-DRI-EAF route in which CO2 emissions from shaft furnace and natural gas combustion are captured using post-combustion amine-based CCS solution. | 1t steel= 1.3t DRI = 1.9t Pellet | 2020 |
| A hydrogen-based direct reduced iron-electric arc furnace route fully replacing natural gas with green hydrogen. | pellet-DRI-EAF | DRI-EAF 100% H_2_ | Pellet-DRI-EAF route in which natural gas is replaced with green hydrogen as reductant. | 1t steel= 1.3t DRI = 1.9t Pellet | 2025 |
| A transitional direct reduced iron-electric arc furnace pathway substituting half of the natural gas with hydrogen. | pellet-DRI-EAF | DRI-EAF 50% H_2_ | Pellet-DRI-EAF route in which 50% of shaft furnace natural gas feed is replaced with green hydrogen. | 1t steel= 1.3t DRI = 1.9t Pellet | 2028 |
| A less conventional route where direct reduced iron is used as feedstock for basic oxygen furnaces. | - | DRI-BOF | Pelletized iron ore is reduced to DRI in a shaft furnace, where natural gas, reformed into a mixture of CO and H_2_, serves as the reductant, replacing coal typically used in blast furnace routes. The resulting DRI is then fed into BOF where it undergoes oxygen treatment similar to BF-BOF route. The scrap ratio is up to 30% as BAT BF-BOF. | 1t steel= 0.8t DRI + 0.3t scrap= 1.2t Pellet | 2026 |
| A hydrogen-based direct reduced iron pathway feeding basic oxygen furnaces, achieving full fossil-free reduction. | pellet-DRI-BOF | DRI-BOF 100% H_2_ | Pellet-DRI-BOF route in which natural gas is replaced with green hydrogen as reductant. | 1t steel= 0.8t DRI + 0.3t scrap= 1.2t Pellet | 2028 |
| A direct reduced iron–basic oxygen furnaces configuration with integrated CCS to curb process carbon emissions. | pellet-DRI-BOF | DRI-BOF CCS | Pellet-DRI-BOF route in which CO2 emissions resulting from all major processes are assumed to be captured using post-combustion amine-based CCS solution. | 1t steel= 0.8t DRI + 0.3t scrap= 1.2t Pellet | 2026 |
| A smelting reduction–basic oxygen furnaces route where iron ore pellets are reduced in a coal-based SR unit before BOF refining. | - | SR-BOF | This is a type of process in which liquid hot metal is produced directly from iron ore without the use of coke. The business case is based on HIsarna, a smelting reduction technology in which uniformly sized, appropriately porous iron ore pellets are injected into the top of a Cyclone Converter Furnace along with pure oxygen, while pulverized coal is supplied at the bottom. The resulting pig iron is transferred to a BOF for decarburization, following a procedure similar to the conventional BF–BOF route. The scrap ratio is assumed to match that of the BAT BF–BOF configuration (30%), and coal consumption is estimated at 750 kg per ton of hot metal. | 1t steel= 0.8t SR+0.3t scrap = 1.2t Pellet + 0.6 t coal | 2020 |
| A smelting reduction–basic oxygen furnaces route is equipped with CCS. | Pellet-SR-BOF | SR-BOF CCS | Pellet-SR-BOF that takes advantage of the fact that CO_2_ emissions from Cyclone Converter Furnace exit as concentrated stream (85-95% CO_2_) which facilitates carbon capture. CCS technique used in the modelling is cryogenic distillation in which the CO2-rich stream is liquefied and split into main constituents via distillation. | 1t steel= 0.8t SR+0.3t scrap = 1.2t Pellet + 0.6 t coal | 2028 |

**Table S4.** EFs of PCDD/Fs, PCBs, HCB, PECBz, PCNs and HCBD for complete steel production routes

| Routes | PCDD/Fs (µg TEQ·t^-1^) | PCBs(µg TEQ·t^-1^) | HCB(µg·t^-1^) | PeCBz(µg·t^-1^) | PCNs (µg TEQ·t^-1^) | HCBD(µg·t^-1^) |
| --- | --- | --- | --- | --- | --- | --- |
| BF-BOF | 0.396-4.777 (1.254) | 0.107-0.620 (0.155) | 287-769 (527) | 1624-2119 (1855) | 0.003-0.021 (0.008) | 0.884-3.208 (1.457) |
| BAT BF-BOF | 0.342-3.982 (1.079) | 0.105-0.523 (0.144) | 231-785 (429) | 1320-1722 (1507) | 0.003-0.018 (0.007) | 0.741-2.629 (1.206) |
| BAT BF-BOF H2 | 0.340-3.981 (1.078) | 0.105-0.523 (0.144) | 231-785 (429) | 1320-1722 (1507) | 0.003-0.018 (0.007) | 0.741-2.629 (1.206) |
| BAT BF-BOF BIO | 0.342-4.020 (1.098) | 0.105-0.527 (0.146) | 231-789 (431) | 1320-1722 (1507) | 0.003-0.018 (0.007) | 0.741-2.629 (1.206) |
| EAF(Scrap) | 0.522-3.666 (1.384) | 0.053-0.564 (0.173) | 177-600 (327) | 1200 | 0.020-0.031 (0.026) | 0.043-3.717 (0.401) |
| Elw-EAF | 0.000 | 0.000 | 0 | 0 | 0 | 0 |
| Ele-EAF | 0.000 | 0.000 | 0 | 0 | 0 | 0 |
| DRI-EAF CCS | 0.228 | 0.000 | 57 | 0 | 0 | 0 |
| DRI-EAF 100% H2 | 0.228 | 0.000 | 57 | 0 | 0 | 0 |
| DRI-EAF 50% H2 | 0.228 | 0.000 | 57 | 0 | 0 | 0 |
| DRI-EAF | 0.228 | 0.000 | 57 | 0 | 0 | 0 |
| DRI-BOF | 0.244-0.324 (0.284) | 0.001-0.002 (0.001) | 38-40(39) | 0 | 0.001-0.002 (0.001) | 0 |
| DRI-BOF 100% H2 | 0.244-0.324 (0.284) | 0.001-0.002 (0.001) | 38-40(39) | 0 | 0.001-0.002 (0.001) | 0 |
| DRI-BOF CCS | 0.244-0.324 (0.284) | 0.001-0.002 (0.001) | 38-40(39) | 0 | 0.001-0.002 (0.001) | 0 |
| SR-BOF | 0.252-0.338 (0.295) | 0.002-0.003 (0.003) | 39-41(40) | 0 | 0.001-0.002 (0.001) | 0 |
| SR-BOF CCS | 0.252-0.338 (0.295) | 0.002-0.003 (0.003) | 39-41(40) | 0 | 0.001-0.002 (0.001) | 0 |

**Table S5. EFs of CO_2_ for different steelmaking routes^[34]^**

| Steelmaking Routes | EFs of CO_2_ (t/t steel) |
| --- | --- |
| BF-BOF | 2.6 |
| BAT BF-BOF | 2 |
| BAT BF-BOF H_2_ | 1.8 |
| BAT BF-BOF BIO | 1.6 |
| EAF(Scrap) | 0.2 |
| Elw-EAF | 0.1 |
| Ele-EAF | 0.1 |
| DRI-EAF | 0.1 |
| DRI-EAF CCS | 0.1 |
| DRI-EAF 100% H_2_ | 0.5 |
| DRI-EAF 50% H_2_ | 0.8 |
| DRI-BOF | 0.7 |
| DRI-BOF 100% H_2_ | 0.1 |
| DRI-BOF CCS | 0.1 |
| SR-BOF | 1.1 |
| SR-BOF CCS | 0.1 |

**Table S6. Chlorine content in reductant in BF^[36, 37]^**

| Countries | Cl in coal | CI in coke | CI in biochar |
| --- | --- | --- | --- |
| Russia | 0.012% | 0.007% | 0.44% |
| Australia | 0.039% | 0.011% |  |
| Australia | 0.055% | 0.009% |  |
| Canada | 0.016% | 0.007% |  |
| China | 0.104% | 0.009% |  |
| Australia | 0.021% | 0.010% |  |
| Canada | 0.029% | 0.016% |  |
| Australia | 0.047% | 0.018% |  |
| Canada | 0.042% | 0.018% |  |
| Canada | 0.012% | 0.004% |  |
| USA | 0.150% | 0.025% |  |
| South Africa | 0.018% | 0.009% |  |
| China | 0.052% | 0.032% |  |
| Australia | 0.036% | 0.020% |  |
| Australia | 0.020% | 0.009% |  |

**Table S7. Comparison of major roadmap scenarios for global steelmaking under the climate goal**

| Criteria / Roadmap | IEA (2020/2021)^[35]^ | MPP (2022)^[34]^ | NewClimate (2022)^[63]^ | Deloitte (2022)^[64]^ | Yu et al. (2021)^[65]^ |
| --- | --- | --- | --- | --- | --- |
| Type of organization | Intergovernmental (IEA) | Multi-stakeholder coalition (industry, policy, consultancy) | Independent research institute | Private consultancy | Academic research group |
| Modeling approach | Full energy-industry system modeling (e.g., TIMES) | Cost-pathway and technology deployment system model | Progress tracking, non-predictive | Hydrogen cost-based sensitivity analysis | Facility-level production + emission modeling |
| Time horizon | 2020–2050 | 2020–2050 | Primarily near/mid-term; limited to 2050 | Targets 2030 and 2050 | To 2050 |
| System boundary | Full lifecycle of global steel production (mine-to-market) | Covers the full value chain + investment and market mechanisms | Tracks national/industry-level policy implementation and transparency | Focuses on corporate decision-making under different cost scenarios | Limited to demand evolution in developing regions |
| Technology resolution | High: differentiated modeling of BF-BOF, DRI, CCUS, EAF, electrolysis, with TRLs, cost, deployment | Very high: 20 archetypes with TRL stages, cost, scalability profiles | Moderate: general tracking of EAF and DRI expansion without stagewise development | Medium: focused on DRI and H2 cost; excludes CCUS/electrolysis pathways | Low: applies static average emission factors to facilities without breakdown |
| Economic modeling detail | Includes energy price, material input, and carbon cost variation under policies | Quantifies TCO, CapEx, O&M by technology; enables policy and investment simulation | No cost modeling or techno-economic comparison | Assesses impact of hydrogen price on H2-DRI feasibility; omits CCUS/electricity/policy costs | No economic modeling; purely scenario-based volume estimation |
| Transparency of assumptions | Fully disclosed: model structure, parameters, geographic scope, system boundary | Clear: covers uptake rates, mitigation %, hydrogen/electricity cost assumptions | Methodology not disclosed; lacks input traceability | Partial: based on corporate data; lacks full parameter disclosure | Static assumptions; lacks generalizability across systems or countries |
| Paris agreement alignment | Explicitly aligned; NZE2050 cited in IPCC pathways | Explicitly aligned; NZE2050 cited in IPCC pathways | Not explicitly stated; aligned with pledges, not climate budgets | Not aligned with temperature goals; cost focus only | No reference to global temperature alignment |
| Output metrics | CO₂ intensity by process (tCO₂/t steel), energy use (GJ/t), electricity share by pathway | Pathway-specific CO2 factors, H2 proportion, energy breakdown available | No process-level emission or energy factors; qualitative tracking only | Provides H2-DRI CO2 abatement estimates (70–95%); lacks broader tech set | General factors; lacks energy structure or technological differentiation |
| Relevance to this study | Highly relevant: provides quantitative projections for emissions, production, and technology shares | Highly relevant: provides quantitative projections for emissions, production, and technology shares | Not suitable: lacks forward-looking modeling or quantitative projections | Not suitable: cost-focused, lacks system structure and mitigation diversity | Not suitable: low resolution, regional focus only, no pathway-based modeling |
| Included in this study | Yes | Yes | No | No | No |

**Table S8. Comparison of scenarios involved in global UPOPs emission model**

| Scenario | Source | Production Assumption | Scenario Description | Emission Constraint | Technology Evolution | Technology Expectation | Production (Gt) | Regulatory Mechanism |
| --- | --- | --- | --- | --- | --- | --- | --- | --- |
| Base | IEA | Business as Usual | This scenario reflects the continuation of existing and announced national policy commitments without additional efforts to decarbonize the steel sector. It assumes no new technological mandates, no carbon pricing, and no coordinated industrial transformation beyond the current trajectory. | 2°C pathway | Economic replacement | Low technology expectation: no significant deployment of breakthrough technologies is expected. Steelmaking routes from 2019-2050 and percentage in 2050 include BF-BOF (36%), EAF (52%), DRI-EAF (11%) and SR-BOF (0%). | 2.54 | - |
| SDS_LTE | IEA | Material Efficiency Strategies | Aligned with the Paris Agreement, the SDS_LTE outlines a climate-compatible pathway for the global steel industry with global warming under 1.7 °C around 2050. This scenario relies on three pillars: (i) significant material efficiency improvements that reduce demand for crude steel by up to 20% compared to Base, (ii) broad deployment of relatively mature low-emissions technologies including CCS and hydrogen-based DRI, and (iii) a transition towards electrification and green hydrogen enabled by a doubling of electricity use. | well-below-2°C pathway | Economic replacement | Moderate technology expectation: The scenario deliberately avoids relying on speculative breakthroughs, focusing instead on accelerated scaling of relatively mature low-carbon options. Steelmaking routes from 2019-2050 and percentage in 2050 include BF-BOF (30%), BAT BF-BOF H_2_(3%), EAF(Scarp) (38%), DRI-EAF(9%), DRI-EAF CCS (2%), DRI-EAF H_2_(8%) and SR-BOF(0%), SR-BOF CCS(10%). | 2.07 | A coordinated set of enabling policies—such as green public procurement, technology deployment incentives, and material efficiency mandates—to support deep decarbonization |
| Base_HTE | MPP | Business as Usual | This reference scenario models a future in which investment decisions are based solely on total cost of ownership (TCO), without any carbon constraints or coordinated decarbonisation policy. While not strictly a business-as-usual case, it assumes only the natural uptake of low-emissions technologies when economically viable. No specific emissions reduction targets or technology bans are enforced. | 2°C pathway | Economic replacement | High technology expectation: the scenario adopts a more ambitious stance, anticipating earlier commercialization and widespread deployment of currently pre-commercial technologies. Steelmaking routes from 2019-2050 and percentage in 2050 include BF-BOF(0%), BAT BF-BOF(1%), BAT BF-BOF H_2_(42%), EAF(Scarp) (35%), DRI-EAF(6%), DRI-BOF(2%), SR-BOF(14%). | 2.54 | - |
| TM_HTE | MPP | Business as Usual | In this scenario, a regulatory moratorium is assumed to take effect from 2030, banning new investments in conventional high-emissions technologies. Before 2030, the technology choice follows the Baseline. After 2030, only (near-) zero-emissions technologies are permitted, including green hydrogen-based DRI, smelting reduction with CCUS, and electrolytic steelmaking. This scenario achieves net-zero emissions by 2050 through investment restrictions alone. | well-below-2°C pathway | Economic replacement | High technology expectation: the scenario adopts a more ambitious stance, anticipating earlier commercialization and widespread deployment of currently pre-commercial technologies. Steelmaking routes from 2019-2050 and percentage in 2050 include BF-BOF(0%), BAT BF-BOF(0%), BAT BF-BOF H_2_ (0%), BAT BF-BOF BIO (1%), BAT BF-BOF CCS (5%), SR-BOF(15%), DRI-BOF(17%), DRI-EAF(12%), EAF(39%), Ele-EAF and Elw-EAF(11% Ele-EAF+ Elw-EAF ). | 2.54 | After 2030, no new investments are permitted in conventional high-emission steelmaking routes. All new capacity must use near-zero-emissions technologies. |
| CC_HTE | MPP | Business as Usual | This scenario explores a carbon-cost-driven transition, applying a steadily rising carbon price—starting at $9/tCO2 in 2023 and reaching $250/tCO2 by 2050—across all emission scopes and geographies. Carbon pricing serves as a proxy for a coordinated suite of policies (e.g., green procurement, subsidies, emissions standards) to internalize the environmental cost of high-emissions production. Earlier shifts to low-CO2 technologies occur due to improved competitiveness. This scenario achieves net-zero emissions by 2050 through carbon price alone. | well-below-2°C pathway | Economic replacement | High technology expectation: the scenario adopts a more ambitious stance, anticipating earlier commercialization and widespread deployment of currently pre-commercial technologies. Steelmaking routes include BF-BOF(0%), BAT BF-BOF(0%), BAT BF-BOF H_2_(0%), BAT BF-BOF BIO(0%), BAT BF-BOF CCS(6%), SR-BOF(7%), DRI-BOF(23%), DRI-EAF(19%), EAF(40%), Ele-EAF and Elw-EAF (6% Ele-EAF+ Elw-EAF). | 2.54 | The Carbon Cost scenario applies a rising carbon price reaching $250/tCO2 by 2050 to simulate a coordinated suite of policy levers that drive early switching to low-carbon technologies. |

**Table S9. Parameters used in health risk model** ^[28, 66, 67, 68, 69, 70, 71, 72, 73, 74, 75, 76, 77, 78, 79, 80, 81, 82, 83]^

| UPOPs | f_g_ | f_g_'1.5^a^ | f_g_'2^b^ | V_g_ (m/s) | V_p_ (m/s) | Halftime (days) | Inhalation Unit Riks^c^ (μg/m^3^)^-1^ | H (m) | H'1.5(m) | H'2(m) |
| --- | --- | --- | --- | --- | --- | --- | --- | --- | --- | --- |
| PCDD/Fs | 0.5(0.3-0.72) | 0.54 (0.33–0.76) | 0.56(0.34-0.77) | 0.00001 | 0.001(0.0004-0.006) | 302(54-550) | 38^a^ | 500(450-800) | 510(459-820) | 515(464-824) |
| PCBs | 0.89(0.80-0.98) | 0.91 (0.82–0.98) | 0.91 (0.83–0.99) |  |  | 82(54-110) |  |  |  |  |
| PCNs | 0.95(0.90-1) | 0.96 (0.91–1.000) | 0.96 (0.91–1.000) |  |  | 6.5(6-7) |  |  |  |  |
| HCB | 0.975(0.95-0.99) | 0.98 (0.96–0.99) | 0.98 (0.96–0.99) |  |  | 770 (156-1570) | 0.00051 |  |  |  |
| HCBD | 0.98 | 0.98 | 0.98 |  |  | 577(60-1095) | 0.000022 |  |  |  |

a: value after applying the +1.5 °C perturbation; b: value after applying the +2 °C perturbation; c: expressed as Inhalation Unit Riks of TEQ;

Data S1 (separate file). The resulting uncertainty ranges for global emissions of UPOPs under all scenarios

Data S2 (separate file). The resulting uncertainty ranges for global emission compositions of steelmaking routes for UPOPs under all scenarios

Data S3 (separate file). The resulting uncertainty ranges for regional emissions of UPOPs under all scenarios

Data S4 (separate file). The resulting uncertainty ranges for regional emission compositions of steelmaking routes for UPOPs under all scenarios

Data S5 (separate file). Population size and life expectancy for a given age group in a specific region

Data S6 (separate file). The resulting uncertainty ranges for regional lifetime inhalation cancer risks

**SI References**

[1] K. Ballschmiter, I. Braunmiller, R. Niemczyk, M. Swerev, Reaction pathways for the formation of polychloro-dibenzodioxins (PCDD) and —dibenzofurans (PCDF) in combustion processes: II. Chlorobenzenes and chlorophenols as precursors in the formation of polychloro-dibenzodioxins and —dibenzofurans in flame chemistry, *Chemosphere* **1988**, *17* (5), 995, <https://doi.org/https://doi.org/10.1016/0045-6535(88)90070-7>.

[2] Q. Yang, L. Yang, J. Shen, Y. Yang, M. Wang, X. Liu, X. Shen, C. Li, J. Xu, F. Li, D. Li, G. Liu, M. Zheng, Polychlorinated dibenzo-p-dioxins and dibenzofurans (PCDD/Fs) emissions from electric arc furnaces for steelmaking, *Emerging Contaminants* **2020**, *6*, 330, <https://doi.org/10.1016/j.emcon.2020.08.005>.

[3] European Commission, Best Available Techniques (BAT) Reference Document for Iron and Steel Production **2013**.

[4] UNEP, Guidelines on best available techniques and provisional guidance on best environmental practices relevant to article 5 and annex c of the stockholm convention on persistent organic pollutants. UNEP: **2007**.

[5] D. S. Drage, E. Aries, S. Harrad, Studies into the formation of PBDEs and PBDD/Fs in the iron ore sintering process, *Sci Total Environ* **2014**, *485-486*, 497, <https://doi.org/10.1016/j.scitotenv.2014.03.093>.

[6] E. Aries, D. R. Anderson, R. Fisher, T. A. Fray, D. Hemfrey, PCDD/F and "Dioxin-like" PCB emissions from iron ore sintering plants in the UK, *Chemosphere* **2006**, *65* (9), 1470, <https://doi.org/10.1016/j.chemosphere.2006.04.020>.

[7] S. Li, G. Liu, M. Zheng, W. Liu, J. Li, M. Wang, C. Li, Y. Chen, Unintentional production of persistent chlorinated and brominated organic pollutants during iron ore sintering processes, *J Hazard Mater* **2017**, *331*, 63, <https://doi.org/10.1016/j.jhazmat.2017.02.027>.

[8] G. Liu, W. Liu, Z. Cai, M. Zheng, Concentrations, profiles, and emission factors of unintentionally produced persistent organic pollutants in fly ash from coking processes, *J Hazard Mater* **2013**, *261*, 421, <https://doi.org/10.1016/j.jhazmat.2013.07.063>.

[9] G. Liu, M. Zheng, P. Lv, W. Liu, C. Wang, B. Zhang, K. Xiao, Estimation and Characterization of Polychlorinated Naphthalene Emission from Coking Industries, *Environmental Science & Technology* **2010**, *44* (21), 8156, <https://doi.org/10.1021/es102474w>.

[10] G. Liu, M. Zheng, B. Du, Z. Nie, B. Zhang, J. Hu, K. Xiao, Identification and characterization of the atmospheric emission of polychlorinated naphthalenes from electric arc furnaces, *Environ Sci Pollut Res Int* **2012**, *19* (8), 3645, <https://doi.org/10.1007/s11356-012-1038-2>.

[11] W. S. Lee, G. P. Chang-Chien, L. C. Wang, W. J. Lee, K. Y. Wu, P. J. Tsai, Emissions of polychlorinated dibenzo-p-dioxins and dibenzofurans from stack gases of electric arc furnaces and secondary aluminum smelters, *J Air Waste Manag Assoc* **2005**, *55* (2), 219, <https://doi.org/10.1080/10473289.2005.10464613>.

[12] L. Stieglitz, Selected Topics on the De Novo Synthesis of PCDD/PCDF on Fly Ash, *Environmental Engineering Science* **1998**, *15* (1), 5, <https://doi.org/10.1089/ees.1998.15.5>.

[13] E. A. Hogendoorn, Y. Bruinen de Bruin, M. P. M. Janssen, Formation of Polychlorinated Biphenyls on Secondary Copper Production Fly Ash: Mechanistic Aspects and Correlation to Other Persistent Organic Pollutants. **2009**.

[14] N. Watanabe, A. Takakura, Y. Minami, S. Mizutani, H. Takatsuki, Correlation of low-volatile organic chlorine (LVOCl) and PCDD/Fs in various municipal waste incinerators (MWIs), *Chemosphere* **2007**, *67* (9), S198, <https://doi.org/10.1016/j.chemosphere.2006.05.100>.

[15] W. Jiang, Y. Peng, M. Tang, S. Xiong, K. Chen, S. Lu, F. Wang, PCDD/Fs emissions in a large-scale hazardous waste incinerator under different operation conditions: Effective reduction strategies and an applicable correlation, *Journal of the Energy Institute* **2023**, *107*, <https://doi.org/10.1016/j.joei.2023.101186>.

[16] V. M. Thomas, C. M. McCreight, Relation of chlorine, copper and sulphur to dioxin emission factors, *J Hazard Mater* **2008**, *151* (1), 164, <https://doi.org/10.1016/j.jhazmat.2007.05.062>.

[17] B. R. Stanmore, Modeling the formation of PCDD/F in solid waste incinerators, *Chemosphere* **2002**, *47*, 565, <https://doi.org/https://doi.org/10.1016/S0045-6535(02)00005-X>.

[18] A. Buekens, L. Stieglitz, K. Hell, H. Huang, P. Segers, Dioxins from thermal and metallurgical processes: recent studies for the iron and steel industry, *Chemosphere* **2001**, *42* (5), 729, <https://doi.org/https://doi.org/10.1016/S0045-6535(00)00247-2>.

[19] J. Liao, A. Buekens, K. Olie, J. Yang, T. Chen, X. Li, Iron and copper catalysis of PCDD/F formation, *Environ Sci Pollut Res Int* **2016**, *23* (3), 2415, <https://doi.org/10.1007/s11356-015-5437-z>.

[20] D. Wang, H. Zhang, M. Ren, Y. Fan, Y. Gao, Z. Y. Lv, Y. Yu, J. Chen, Electrophilic chlorination of dibenzo-p-dioxin and dibenzofuran over composite copper and iron chlorides and oxides in combustion flue gas, *Chemosphere* **2020**, *256*, 127065, <https://doi.org/10.1016/j.chemosphere.2020.127065>.

[21] J. Y. Ryu, Formation of chlorinated phenols, dibenzo-p-dioxins, dibenzofurans, benzenes, benzoquinnones and perchloroethylenes from phenols in oxidative and copper (II) chloride-catalyzed thermal process, *Chemosphere* **2008**, *71* (6), 1100, <https://doi.org/10.1016/j.chemosphere.2007.10.036>.

[22] J. Chen, J. Avise, B. Lamb, E. Salathé, C. Mass, A. Guenther, C. Wiedinmyer, J. F. Lamarque, S. O'Neill, D. McKenzie, N. Larkin, The effects of global changes upon regional ozone pollution in the United States, *Atmos. Chem. Phys.* **2009**, *9* (4), 1125, <https://doi.org/10.5194/acp-9-1125-2009>.

[23] A. Hu, X. Xie, K. Gong, Y. Hou, Z. Zhao, J. Hu, Assessing the Impacts of Climate Change on Meteorology and Air Stagnation in China Using a Dynamical Downscaling Method, *Frontiers in Environmental Science* **2022**, *Volume 10 - 2022*, <https://doi.org/10.3389/fenvs.2022.894887>.

[24] T. Harner, N. J. L. Green, K. C. Jones, Measurements of Octanol−Air Partition Coefficients for PCDD/Fs:  A Tool in Assessing Air−Soil Equilibrium Status, *Environmental Science & Technology* **2000**, *34* (15), 3109, <https://doi.org/10.1021/es000970m>.

[25] J. F. Pankow, An absorption model of gas/particle partitioning of organic compounds in the atmosphere, *Atmospheric Environment* **1994**, *28* (2), 185, <https://doi.org/https://doi.org/10.1016/1352-2310(94)90093-0>.

[26] OEHHA, Appendix E Determination of Chemicals for Multipathway Analysis. **2012**.

[27] M. L. Wesely, Parameterization of surface resistances to gaseous dry deposition in regional-scale numerical models, *Atmospheric Environment* **2007**, *41*, 52, <https://doi.org/https://doi.org/10.1016/j.atmosenv.2007.10.058>.

[28] San Francisco Estuary Institute, Estimated Atmospheric Deposition Fluxes of Dioxins in the San Francisco Estuary. **2012**.

[29] NASA, Air Viscosity, Sutherland's Formula. **2009**.

[30] M. L. Laucks, Aerosol Technology Properties, Behavior, and Measurement of Airborne Particles: William C. Hinds. Wiley, New York (1999). ISBN 0-471-19410-7. 464 pages+Index, *Journal of Aerosol Science* **2000**, *31* (9), 1121, <https://doi.org/https://doi.org/10.1016/S0021-8502(99)00571-6>.

[31] W. W. Brubaker, R. A. Hites, OH Reaction Kinetics of Polycyclic Aromatic Hydrocarbons and Polychlorinated Dibenzo-p-dioxins and Dibenzofurans, *The Journal of Physical Chemistry A* **1998**, *102* (6), 915, <https://doi.org/10.1021/jp9721199>.

[32] C. Yan, J. Chen, L. Huang, G. Ding, X. Huang, Linear free energy relationships on rate constants for the gas-phase reactions of hydroxyl radicals with PAHs and PCDD/Fs, *Chemosphere* **2005**, *61* (10), 1523, <https://doi.org/https://doi.org/10.1016/j.chemosphere.2005.04.037>.

[33] P. H. Taylor, T. Yamada, A. Neuforth, Kinetics of OH radical reactions with dibenzo-p-dioxin and selected chlorinated dibenzo-p-dioxins, *Chemosphere* **2005**, *58* (3), 243, <https://doi.org/https://doi.org/10.1016/j.chemosphere.2004.07.054>.

[34] Mission Possible Partnership, The Net Zero Steel Sector Transition Strategy. **2021**.

[35] IEA, Iron and Steel Technology Roadmap. **2020**.

[36] S. Nomura, Behavior of coal chlorine in cokemaking process, *International Journal of Coal Geology* **2010**, *83* (4), 423, <https://doi.org/10.1016/j.coal.2010.06.003>.

[37] K. Wilsona, D. Reed IBI White Paper-Implications and Risks of Potential Dioxin Presence in Biochar. International Biochar Initiative,,: **2012**.

[38] WHO, Steel-Raw materials. **2025**.

[39] S. Sanjal, The Value of DRI – Using the Product for Optimum Steelmaking. **2015**.

[40] UNEP, Smelt reduction for iron and steel sector. **2025**.

[41] T. Battle, U. Srivastava, J. Kopfle, R. Hunter, J. McClelland, in (Eds.: S. Seetharaman, R. Guthrie, A. McLean, S. Seetharaman, H. Y. Sohn), Elsevier, **2024**.

[42] C. Li, G. Liu, S. Qin, T. Zhu, J. Song, W. Xu, Emission reduction of PCDD/Fs by flue gas recirculation and activated carbon in the iron ore sintering, *Environ Pollut* **2023**, *327*, 121520, <https://doi.org/10.1016/j.envpol.2023.121520>.

[43] X. Liu, M. Ye, X. Wang, W. Liu, T. Zhu, Gas-phase and particle-phase PCDD/F congener distributions in the flue gas from an iron ore sintering plant, *J Environ Sci (China)* **2017**, *54*, 239, <https://doi.org/10.1016/j.jes.2016.01.023>.

[44] B. Huang, M. Gan, Z. Ji, X. Fan, G. Wang, Z. Sun, Q. Zhao, Y. Wu, S. Lu, Co-treating MSWI fly ash in iron ore sintering process: Influence of water-washing and roll forming pretreatment on dioxins emission, *Process Safety and Environmental Protection* **2023**, *173*, 143, <https://doi.org/10.1016/j.psep.2023.03.021>.

[45] Z. Ji, B. Huang, M. Gan, X. Fan, G. Wang, Q. Zhao, J. Xing, R. Yuan, Dioxins control as co-processing water-washed municipal solid waste incineration fly ash in iron ore sintering process, *J Hazard Mater* **2022**, *423* (Pt B), 127138, <https://doi.org/10.1016/j.jhazmat.2021.127138>.

[46] H. He, S. Lu, Y. Peng, M. Tang, M. Zhan, S. Lu, L. Xu, W. Zhong, L. Xu, Emission characteristics of dioxins during iron ore Co-sintering with municipal solid waste incinerator fly ash in a sintering pot, *Chemosphere* **2022**, *287* (Pt 1), 131884, <https://doi.org/10.1016/j.chemosphere.2021.131884>.

[47] Y.-C. Chen, Y.-C. Kuo, M.-R. Chen, Y.-F. Wang, C.-H. Chen, M.-Y. Lin, C. Yoon, P.-J. Tsai, Reducing polychlorinated dibenzo-p-dioxins and dibenzofurans (PCDD/F) emissions from a real-scale iron ore sinter plant by adjusting its sinter raw mix, *Journal of Cleaner Production* **2016**, *112*, 1184, <https://doi.org/10.1016/j.jclepro.2015.07.013>.

[48] V. Esposito, A. Maffei, D. Bruno, B. Varvaglione, S. Ficocelli, C. Capoccia, M. Spartera, R. Giua, M. Blonda, G. Assennato, POP emissions from a large sinter plant in Taranto (Italy) over a five-year period following enforcement of new legislation, *Sci Total Environ* **2014**, *491-492*, 118, <https://doi.org/10.1016/j.scitotenv.2014.03.077>.

[49] C. Zou, J. Han, H. Fu, Emissions of PCDD/Fs from Steel and Secondary Nonferrous Productions, *Procedia Environmental Sciences* **2012**, *16*, 279, <https://doi.org/10.1016/j.proenv.2012.10.039>.

[50] S. Song, X. Zhou, C. Guo, H. Zhang, T. Zeng, Y. Xie, J. Liu, C. Zhu, X. Sun, Emission characteristics of polychlorinated, polybrominated and mixed polybrominated/chlorinated dibenzo-p-dioxins and dibenzofurans (PCDD/Fs, PBDD/Fs, and PBCDD/Fs) from waste incineration and metallurgical processes in China, *Ecotoxicol Environ Saf* **2019**, *184*, 109608, <https://doi.org/10.1016/j.ecoenv.2019.109608>.

[51] L.-C. Wang, Y.-F. Wang, H.-C. Hsi, G.-P. Chang-Chien, Characterizing the Emissions of Polybrominated Diphenyl Ethers (PBDEs) and Polybrominated Dibenzo-p-dioxins and Dibenzofurans (PBDD/Fs) from Metallurgical Processes, *Environmental Science & Technology* **2010**, *44* (4), 1240, <https://doi.org/10.1021/es903128e>.

[52] G. Liu, M. Zheng, B. Du, Z. Nie, B. Zhang, W. Liu, C. Li, J. Hu, Atmospheric emission of polychlorinated naphthalenes from iron ore sintering processes, *Chemosphere* **2012**, *89* (4), 467, <https://doi.org/10.1016/j.chemosphere.2012.05.101>.

[53] C. Zhao, L. Yang, Y. Sun, C. Chen, Z. Huang, Q. Yang, J. Yun, A. Habib, G. Liu, M. Zheng, G. Jiang, Atmospheric emissions of hexachlorobutadiene in fine particulate matter from industrial sources, *Nat Commun* **2024**, *15* (1), 4737, <https://doi.org/10.1038/s41467-024-49097-0>.

[54] EEA, EMEP/EEA air pollutant emission inventory guidebook 2023. EEA: **2023**.

[55] J.-C. Chiu, Y.-H. Shen, H.-W. Li, L.-F. Lin, L.-C. Wang, G.-P. Chang-Chien, Emissions of Polychlorinated Dibenzo-p-dioxins and Dibenzofurans from an Electric Arc Furnace, Secondary Aluminum Smelter, Crematory and Joss Paper Incinerators, *Aerosol and Air Quality Research* **2011**, *11* (1), 13, <https://doi.org/10.4209/aaqr.2010.06.0051>.

[56] M. T. N. Pham, H. Q. Anh, X. T. Nghiem, B. M. Tu, T. N. Dao, M. H. Nguyen, Characterization of PCDD/Fs and dioxin-like PCBs in flue gas from thermal industrial processes in Vietnam: A comprehensive investigation on emission profiles and levels, *Chemosphere* **2019**, *225*, 238, <https://doi.org/10.1016/j.chemosphere.2019.03.024>.

[57] P. Antunes, P. Viana, T. Vinhas, J. Rivera, E. M. Gaspar, Emission profiles of polychlorinated dibenzodioxins, polychlorinated dibenzofurans (PCDD/Fs), dioxin-like PCBs and hexachlorobenzene (HCB) from secondary metallurgy industries in Portugal, *Chemosphere* **2012**, *88* (11), 1332, <https://doi.org/10.1016/j.chemosphere.2012.05.032>.

[58] A. Grochowalski, C. Lassen, M. Holtzer, M. Sadowski, T. Hudyma, Determination of PCDDs, PCDFs, PCBs and HCB emissions from the metallurgical sector in Poland, *Environ Sci Pollut Res Int* **2007**, *14* (5), 326, <https://doi.org/10.1065/espr2006.05.303>.

[59] S. Caserini, A. M. Monguzzi, PCDD/Fs emissions inventory in the Lombardy Region: results and uncertainties, *Chemosphere* **2002**, *48* (8), 779, <https://doi.org/https://doi.org/10.1016/S0045-6535(02)00127-3>.

[60] J. B. Wang, C. H. Hung, C. H. Hung, G. P. Chang-Chien, Polychlorinated dibenzo-p-dioxin and dibenzofuran emissions from an industrial park clustered with metallurgical industries, *J Hazard Mater* **2009**, *161* (2-3), 800, <https://doi.org/10.1016/j.jhazmat.2008.04.026>.

[61] G. Liu, M. Zheng, M. Cai, Z. Nie, B. Zhang, W. Liu, B. Du, S. Dong, J. Hu, K. Xiao, Atmospheric emission of polychlorinated biphenyls from multiple industrial thermal processes, *Chemosphere* **2013**, *90* (9), 2453, <https://doi.org/10.1016/j.chemosphere.2012.11.008>.

[62] M. Yamamoto, K. Kokeguchi, G. Yamamoto, N. Yamaguchi, K. Ohtsuka, S. Sakai, Air emission factors and emission inventory of HCB, PCB and pentachlorobenzene, *Organohalogen Compd.* **2011**, *73*, 388.

[63] NewClimate Institute, Decarbonisation in the global steel sector: tracking the progress **2022**.

[64] Deloitte, Steel-Pathways to decarbonization. London, **2023**.

[65] S. Yu, J. Lehne, N. Blahut, M. Charles, 1.5 C steel: decarbonizing the steel sector in Paris-compatible pathways, *Pacific Northwest National Laboratory* **2021**.

[66] H. Zhao, H. Che, Y. Ma, Y. Wang, H. Yang, Y. Liu, Y. Wang, H. Wang, X. Zhang, The Relationship of PM Variation with Visibility and Mixing-Layer Height under Hazy/Foggy Conditions in the Multi-Cities of Northeast China, *International Journal of Environmental Research and Public Health* **2017**, *14* (5), <https://doi.org/10.3390/ijerph14050471>.

[67] D. Li, Y. Wu, B. Gross, F. Moshary, Dynamics of Mixing Layer Height and Homogeneity from Ceilometer-Measured Aerosol Profiles and Correlation to Ground Level PM2.5 in New York City, *Remote Sensing* **2022**, *14* (24), <https://doi.org/10.3390/rs14246370>.

[68] X. Zhu, G. Tang, J. Guo, B. Hu, T. Song, L. Wang, J. Xin, W. Gao, C. Münkel, K. Schäfer, X. Li, Y. Wang, Mixing layer height on the North China Plain and meteorological evidence of serious air pollution in southern Hebei, *Atmospheric Chemistry and Physics* **2018**, *18* (7), 4897, <https://doi.org/10.5194/acp-18-4897-2018>.

[69] A. Mues, M. Rupakheti, C. Münkel, A. Lauer, H. Bozem, P. Hoor, T. Butler, M. G. Lawrence, Investigation of the mixing layer height derived from ceilometer measurements in the Kathmandu Valley and implications for local air quality, *Atmospheric Chemistry and Physics* **2017**, *17* (13), 8157, <https://doi.org/10.5194/acp-17-8157-2017>.

[70] C. Lotteraner, M. Piringer, Mixing-Height Time Series from Operational Ceilometer Aerosol-Layer Heights, *Boundary-Layer Meteorology* **2016**, *161* (2), 265, <https://doi.org/10.1007/s10546-016-0169-2>.

[71] B. Barbas, A. de la Torre, P. Sanz, I. Navarro, B. Artíñano, M. A. Martínez, Gas/particle partitioning and particle size distribution of PCDD/Fs and PCBs in urban ambient air, *Science of The Total Environment* **2018**, *624*, 170, <https://doi.org/https://doi.org/10.1016/j.scitotenv.2017.12.114>.

[72] D. R. Cortes, I. Basu, C. W. Sweet, K. A. Brice, R. M. Hoff, R. A. Hites, Temporal Trends in Gas-Phase Concentrations of Chlorinated Pesticides Measured at the Shores of the Great Lakes, *Environmental Science & Technology* **1998**, *32* (13), 1920, <https://doi.org/10.1021/es970955q>.

[73] Q. Zhu, X. Zhang, S. Dong, L. Gao, G. Liu, M. Zheng, Gas and particle size distributions of polychlorinated naphthalenes in the atmosphere of Beijing, China, *Environ Pollut* **2016**, *212*, 128, <https://doi.org/10.1016/j.envpol.2016.01.065>.

[74] J. B. Manchester-Neesvig, A. W. Andren, Seasonal variation in the atmospheric concentration of polychlorinated biphenyl congeners, *Environmental Science & Technology* **1989**, *23* (9), 1138, <https://doi.org/10.1021/es00067a012>.

[75] N. T. Thuan, N. D. Dat, N. M. Ngoc, N. T.-T. Hoang, T. T. Hien, M. B. Chang, Atmospheric Polychlorinated Naphthalenes in a Tropical Megalopolis of Vietnam: Concentrations, Potential Sources, and Health Risk, *Aerosol and Air Quality Research* **2024**, *24* (8), 240047, <https://doi.org/10.4209/aaqr.240047>.

[76] T. H. Nøst, A. K. Halse, M. Schlabach, A. Bäcklund, S. Eckhardt, K. Breivik, Low concentrations of persistent organic pollutants (POPs) in air at Cape Verde, *Science of The Total Environment* **2018**, *612*, 129, <https://doi.org/https://doi.org/10.1016/j.scitotenv.2017.08.217>.

[77] J. Castro-Jiménez, S. J. Eisenreich, M. Ghiani, G. Mariani, H. Skejo, G. Umlauf, J. Wollgast, J. M. Zaldívar, N. Berrojalbiz, H. I. Reuter, J. Dachs, Atmospheric Occurrence and Deposition of Polychlorinated Dibenzo-p-Dioxins and Dibenzofurans (PCDD/Fs) in the Open Mediterranean Sea, *Environmental Science & Technology* **2010**, *44* (14), 5456, <https://doi.org/10.1021/es100718n>.

[78] J. Heo, D. Kim, G. Lee, Congener Profiles and Source-Wise Phase Partitioning Analysis of PCDDs/Fs and PCBs in Gyeonggi-Do Ambient Air, South Korea. In *International Journal of Environmental Research and Public Health*, **2014**; Vol. 11, pp 11065.

[79] A. Saral, G. Gunes, A. Karadeniz, B. I. Goncaloglu, Gas/particle partitioning of PCDD/F compounds in the atmosphere of Istanbul, *Chemosphere* **2015**, *118*, 246, <https://doi.org/https://doi.org/10.1016/j.chemosphere.2014.09.039>.

[80] J. Castro-Jiménez, S. J. Eisenreich, G. Mariani, H. Skejo, G. Umlauf, Monitoring atmospheric levels and deposition of dioxin-like pollutants in sub-alpine Northern Italy, *Atmospheric Environment* **2012**, *56*, 194, <https://doi.org/https://doi.org/10.1016/j.atmosenv.2012.03.081>.

[81] W. Wang, K. Cui, R. Zhao, J. Zhu, Q. Huang, W.-J. Lee, Sensitivity Analysis of PM2.5-Bound Total PCDD/Fs-TEQ Content: In the Case of Wuhu City, China, *Aerosol and Air Quality Research* **2018**, *18* (2), 407, <https://doi.org/10.4209/aaqr.2017.11.0507>.

[82] F. Yu, K. Cui, H.-L. Sheu, Y.-K. Hsieh, X. Tian, Atmospheric Concentration, Particle-bound Content, and Dry Deposition of PCDD/Fs, *Aerosol and Air Quality Research* **2021**, *21* (5), 210059, <https://doi.org/10.4209/aaqr.210059>.

[83] R. Zhao, K. Cui, W. Wang, L.-C. Wang, P. Yan, Atmospheric PM2.5 and total PCDD/Fs-WHO2005-TEQ Level: A Case of Handan and Kaifeng Cities, China, *Aerosol and Air Quality Research* **2018**, *18* (4), 994, <https://doi.org/10.4209/aaqr.2018.02.0040>.
